# Sustainable nickel enabled by hydrogen-based reduction

**DOI:** 10.1038/s41586-025-08901-7

**Published:** 2025-04-30

**Authors:** U. Manzoor, L. Mujica Roncery, D. Raabe, I. R. Souza Filho

**Affiliations:** 1 Max Planck Institute for Sustainable Materials, Düsseldorf, Germany; 2 Universidad Pedagógica y Tecnológica de Colombia, Tunja, Colombia; 3 Institut Jean Lamour, CNRS (UMR 7198), Université de Lorraine, Nancy, France

**Keywords:** Materials science, Metals and alloys

## Abstract

Nickel is a critical element in the shift to sustainable energy systems, with the demand for nickel projected to exceed 6 million tons annually by 2040^1–4^, largely driven by the electrification of the transport sector. Primary nickel production uses acids and carbon-based reductants, emitting about 20 tons of carbon dioxide per ton of nickel produced^5–7^. Here we present a method using fossil-free hydrogen-plasma-based reduction to extract nickel from low-grade ore variants known as laterites. We bypass the traditional multistep process and combine calcination, smelting, reduction and refining into a single metallurgical step conducted in one furnace. This approach produces high-grade ferronickel alloys at fast reduction kinetics. Thermodynamic control of the atmosphere of the furnace enables selective nickel reduction, yielding an alloy with minimal impurities (<0.04 wt% silicon, approximately 0.01 wt% phosphorus and <0.09 wt% calcium), eliminating the need for further refining. The proposed method has the potential to be up to about 18% more energy efficient while cutting direct carbon dioxide emissions by up to 84% compared with current practice. Our work thus shows a sustainable approach to help resolve the contradiction between the beneficial use of nickel in sustainable energy technologies and the environmental harm caused by its production.

## Abstract (web summary)

A one-step process using fossil-free hydrogen-plasma-based reduction is used to extract nickel from low-grade ore and create high-grade ferronickel alloys, providing a more sustainable and environmentally friendly method for obtaining nickel.

## Main

**Fig. 1:**
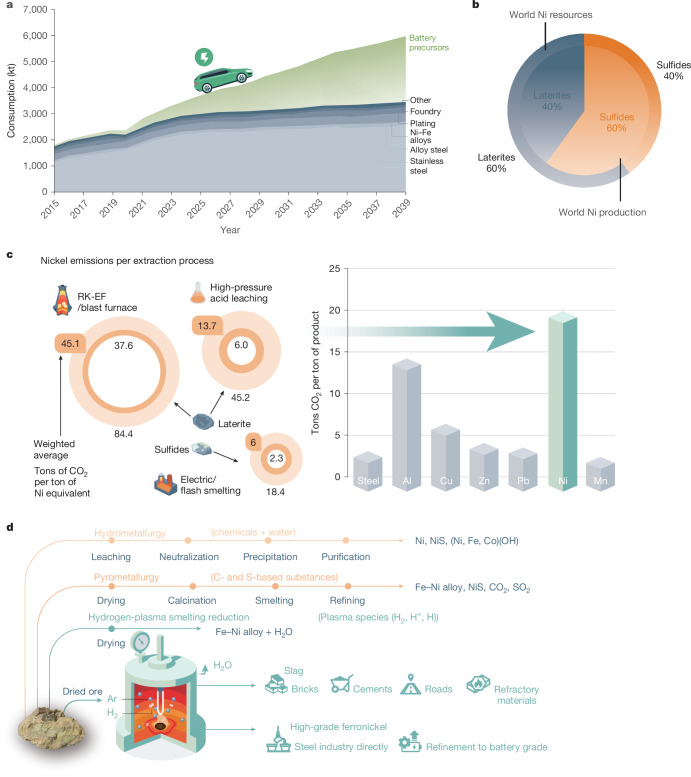
Integrated overview of the production of Ni from its natural ores. The market growth, sources, production, processing routes, emissions and comparison with the sustainable one-step hydrogen-plasma route. **a**, Current and projected (2040) Ni-market growth, fuelled by surging demand for battery electrode precursor materials needed for the electrification of the transport sector, propelling Ni demand to double from 3 million tons to 6 million tons (data from ref. ^1^), at a staggering environmental toll of 20 tons of CO_2_ per ton of Ni (10 times the emission caused by the production of a ton of steel). **b**, Schematic showing the land-based world Ni-resources distribution and their corresponding share in global Ni supply (data from ref. ^8^). **c**, CO_2_ emissions (tons per ton of Ni) from various extraction routes. The inner and outer circles signify lower and upper emission limits. The highlighted middle ring numbers depict weighted averages for each process (data from ref. ^15^). Bar plots on the right show the overall average CO_2_ emissions from the Ni industry (highlighted by green arrow), alongside comparisons with other industries (data from refs. ^5,7^). **d**, Comparative analysis of current processing routes and HPSR: the schematic diagram highlights distinct processing steps for each extraction method. One-step HPSR stands out as a single-step process, directly feeding dried ore into a furnace flooded with an Ar–H_2_ mixture. This streamlined process yields high-grade ferronickel, slag and gaseous water as the products.

Nickel (Ni) is a strategic and hard-to-replace element used in 1.97 million tons of stainless steel and 210 kilotons of non-ferrous alloys, especially superalloys, annually^1–3^. Both alloy families serve sustainability indirectly, the former through enhanced longevity of products and the latter through higher efficiency of engines. Although 70% of the current global annual Ni production (3 million tons)^1–3^ is destined for the stainless-steel sector, the decarbonization of the transport sector through the use of Ni-based battery electrodes in electric vehicles is forecast to require an additional 3 million tons of Ni solely for battery production by 2040. This will cause a doubling of the global Ni demand to 6 million tons per year^1–4^ (Fig. 1a). Currently, 60% of the annual Ni production relies on high-grade sulfide ores (with 1.5–4 wt% Ni content). Low-grade ore variants, namely, laterites (with an average 1.5 wt% Ni content), which are subdivided into two variants, namely, saprolite and limonite, provide the remaining 40% (ref. ^8^; Fig. 1b). However, land-based Ni reserves are inversely distributed, that is, 60% of the total Ni available in nature is found in laterites, and only 40% in sulfide ores^8^ (Fig. 1b).

In sulfidic ore deposits, Ni exists predominantly in the form of discrete binary and ternary Ni-rich minerals such as NiS, Ni_2_FeS_4_ and (Co,Ni)_3_S_4_. The chemical simplicity of sulfide minerals enables effective separation of gangue impurities from valuable Ni-bearing compounds using conventional techniques such as froth flotation^9^. However, the finite and declining reserves of Ni sulfides cannot meet the rapidly increasing global Ni demand, prompting the need for sustainable Ni production from abundant, low-grade laterites. In laterite deposits, Ni is not found as discrete minerals; it is dissolved within complex magnesium (Mg)-silicates (namely, saprolites)^10^, such as (Mg,Fe,Ni)_3_Si_2_O_5_(OH)_4_ and (Mg,Fe,Ni)_3_Si_4_O_10_(OH)_2_.4H_2_O, or partially replaces iron (Fe) in Fe-oxide lattices (limonite)^7^, namely, goethite (Fe,Ni)OOH. This mineralogical and chemical complexity limits the efficient and sustainable beneficiation of these ores into Ni-concentrated substances for downstream green technologies. This fundamental challenge motivated us to develop a completely different approach, namely, a smelting reduction process of the entire dried ore charge in a single metallurgical step using hydrogen plasma. This process integrates calcination, smelting and refining into a single step, with all these operations occurring simultaneously within one furnace, thus, allowing for the direct tapping of high-grade ferronickel from the dried ore charge in just one metallurgical step. A detailed explanation of the term ‘single metallurgical step’ is provided in Supplementary Information section ‘Definition of a single metallurgical step’.

The current industrial Ni-laterite processing routes are largely dictated by the crystallographic structure of Ni-hosting phases and the Ni and Fe content in the ore. Limonite ores, characterized by low Ni and MgO content (<4 wt% Mg), are typically processed via high-pressure acid leaching (HPAL) for Ni and cobalt (Co) recovery, when present^11,12^. In contrast, silicate-rich saprolites, which are difficult to reduce, undergo pyrometallurgical processing. This involves ore drying and partial reduction in rotary kilns (RK) using coke as a reductant, followed by smelting in electric arc furnaces (EAFs), a method known as RK-EF^13,14^, or alternatively in blast furnaces^13^. Further technical details are provided in Supplementary Information section ‘Current industrial processing routes of Ni-laterites’

The energy demand for laterite-ore processing is immense, ranging from 230 GJ per ton of Ni to about 570 GJ per ton of Ni (refs. ^5,6^), far exceeding the 22 GJ per ton required for steel. Greenhouse gas emissions are also substantial, with the RK-EF/blast furnace and HPAL routes emitting 45 tons of carbon dioxide equivalent (CO_2_e) per ton of Ni and 14 tons CO_2_e per ton of Ni, respectively^15^ (Fig. 1c). Although 60% of Ni comes from lower-emission sulfide processing (6 tons CO_2_e per ton of Ni)^15^, laterites account for 40%, pushing the industry’s overall footprint to about 20–27 tons CO_2_e per ton of Ni (refs. ^5–7^; Fig. 1c), over 10 times that of steel (2.3 tons CO_2_e per ton). This makes Ni one of the most environmentally harmful metals to produce (Fig. 1c).

To mitigate emissions, researchers have investigated replacing carbon-based reductants with hydrogen gas (H_2_)^7^ in the solid-state direct reduction of laterites, particularly limonite^7,16,17^. However, the complex crystallographic structure, low Ni content (0.5–2 wt%) and up to 90% impurity contents (oxides of silicon (Si), calcium (Ca) and aluminium (Al) to name a few) hinder reduction, leading to sluggish kinetics^16,18^ and inefficient hydrogen utilization^18,19^, and necessitates several additional pre- and post-treatments^20^ to separate the metal from unreduced oxides. The direct reduction of saprolites with gaseous hydrogen (H_2_) is scarcely reported, probably owing to the thermodynamic stability of silicates^18,21^ at typical direct-reduction temperatures (800–1,000 °C), and its reduction by molecular hydrogen might not be feasible without prior chemical transformation into simpler substances, such as NiO, by using catalyst compounds^22^, as detailed in Supplementary Information section ‘Solid-state DR of laterites’.

**Fig. 2:**
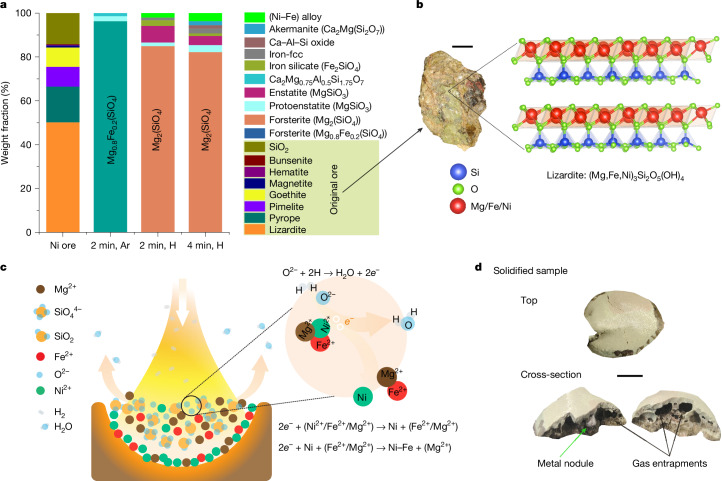
Unveiling phase transformations. The complex Ni-hosting mineral structure in the original ore, reduction mechanisms and a visual snapshot of a solidified sample. **a**, Phase evolution during melting of the original ore in Ar and a lean H_2_ atmosphere (Ar–10% H_2_) for 2 min and 4 min, depicting the transformation of the complex original ore with various phases into simple Mg-silicate phases. The term ‘(Ni–Fe) alloy’ (shown in bright green), represents the metallic nodules, and ‘Iron-fcc’, shown in grey, denotes iron droplets entrapped in silicates that could not be mechanically recovered. **b**, Photograph of the original ore alongside the complex structure of lizardite that is present in it. Lizardite exhibits the capacity to host Ni^2+^/Fe^2+^ ions by replacing Mg^2+^ ions, highlighting the structural intricacies within the ore. **c**, Schematic hypothesis of the reduction mechanism. Precipitation of metals is initiated by the removal of free oxygen from the melt. Hydrogen species extract free oxygen, leaving behind two electrons. These electrons selectively react with Ni^2+^, inducing the precipitation of metallic Ni. The corresponding ionic reactions are also illustrated. **d**, Solidified sample and cross-section. Notably, metallic nodules are observable within the sample, alongside substantial porosities resulting from gas entrapments. Scale bars, 1 cm (**b**) and 5 mm (**d**).

To overcome these limitations, particularly the ones associated with the saprolites, we subject the ore (Extended Data Tables 1 and 2) to a single-step hydrogen-plasma molten state process, thereby dissociating the crystallographic structure of the minerals (for example, (Mg,Fe,Ni)_3_Si_2_O_5_(OH)_4_; Fig. 2b) into simpler ionic species (Fe^2+^, Ni^2+^, Mg^2+^, O^2−^ and SiO_4_^4−^) without the need of catalysts, and concomitantly exposing it to the highly reactive hydrogen-plasma species (H_2_, H and H^+^). This approach consolidates the lengthy and environmentally harmful processes shown in Fig. 1 into a single metallurgical step: hydrogen-plasma smelting reduction (HPSR), beneficiation, metal separation and refining. Operable entirely on renewable energy, it replaces carbon-based fuels and reductants with renewable electricity and hydrogen, offering up to 18% energy savings and a reduction in CO_2_ emissions of up to 84%. Additional plasma-free experiments with molten Ni ore and molecular hydrogen were conducted as reference and comparison tests. The results showed significantly slower reaction kinetics with molecular hydrogen compared with hydrogen plasma, discussed in detail in Supplementary Information section ‘Experiments with molecular hydrogen’.

Our investigation is focused on understanding the hydrogen-plasma reduction behaviour of saprolitic low-grade Ni laterite ore (Extended Data Tables 1 and 2). This approach marks a promising departure from conventional industrial techniques such as RK-EF/blast furnace and HPAL, by replacing carbon (C)- and sulfur (S)-based reducing agents with hydrogen and hence minimizing or eliminating direct CO_2_ and sulfur dioxide (SO_2_) emissions, circumventing the use of harmful acids (for example, sulfuric acid (H_2_SO_4_) in HPAL), and negating the need for costly pre- and post-treatments (Fig. 1d). This study provides experimental evidence supporting one-step HPSR as a sustainable alternative for metal production from both oxides and silicates. This expands feedstock options to low-cost, low-grade minerals. We investigate the thermodynamics, chemical partitioning, microstructural evolution and reaction mechanisms of Ni extraction from lateritic ores, applying these principles for precise and optimal process design.

## Original laterite Ni ore

**Extended Data Fig. 1:**
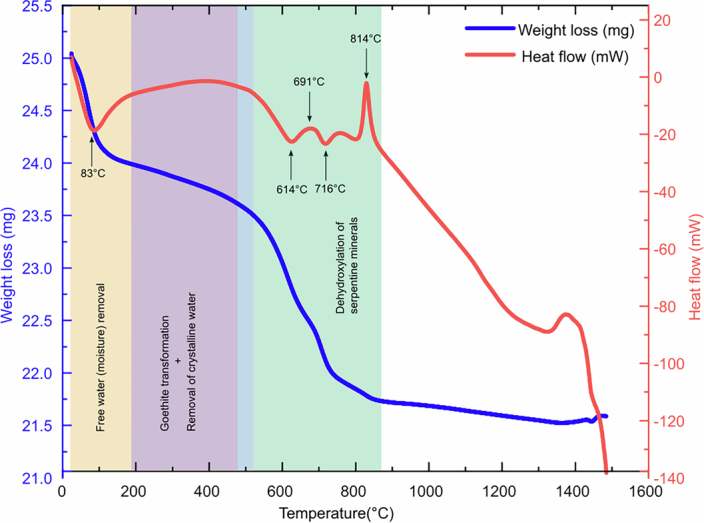
Weight loss of Ni-ore due to thermal decomposition. Weight loss experienced by Ni-Ore upon heating in an inert atmosphere (Ar) as determined by TGA-DSC. The weight loss is broadly categorized into three stages: Stage 1 - weight loss due to the removal of moisture present in the ore. Stage 2 - weight loss attributed to the transformation of goethite into haematite (2FeO(OH) → Fe_2_O_3_ + H_2_O) and the removal of crystalline water from pimelite (Ni_3_Si_4_O_10_(OH)_2_·4H_2_O). Stage 3 - Dehydroxylation of serpentine minerals such as lizardite to forsterite, fayalite, entstantite type species (2Mg_3_Si_2_O_5_(OH)_4_ → 3Mg_2_SiO_4_ + SiO_2_ + 4H_2_O; (Mg,Fe,Ni)_3_Si_2_O_5_(OH)_4_ → 3(Mg,Fe,Ni)_2_SiO_4_ + SiO_2_ + 4H_2_O; (Mg,Fe,Ni)_3_Si_2_O_5_(OH)_4_ → (Mg,Fe,Ni)SiO_3_ + (Mg,Fe,Ni)_2_SiO_4_ + 2H_2_O) – and transformation from olivines to pyroxenes (Mg_2_SiO_4_ + SiO_2_ → 2MgSiO_3_). Endothermic peaks are related with dehydroxylation reactions and decomposition of serpentines to simpler species. The exothermic peak at 814 °C is related to the formation of pyroxene from olivine species, such as enstantite.

The chemical and phase composition of the laterite ore investigated in this study was determined by microwave plasma atomic absorption spectroscopy and X-ray diffraction (XRD), respectively, and are documented in Extended Data Tables 1 and 2. The original ore contains only 1.26 wt% Ni, mainly distributed between the Ni_4_O_4_ (0.4 wt%) and Ni_3_Si_4_O_10_(OH)·4H_2_O (8.02 wt%) constituents. However, we cannot discard the minor solubility of such an element in phases such as lizardite (Mg_3_(SiO_5_)(OH)_4_ (Fig. 2b) and pyrope (Mg_3_Al_2_(SiO_4_)_3_), as also documented in the literature^11,12,23,24^. The as-received ore was compacted into green pellets and smelted for 2 minutes in an EAF under an inert argon (Ar) atmosphere, as described in Methods. The solidified sample showed a weight loss of approximately 23 wt%, attributed to physical losses from thermal decomposition reactions occurring at temperatures above 1,600 °C and low oxygen partial pressures, as in our furnace conditions. The weight loss was further analysed using thermogravimetric analysis–differential scanning calorimetry and the results are shown in Extended Data Fig. 1. The thermogravimetric analysis–differential scanning calorimetry data reveal that the ore’s weight loss up to 1,500 °C is primarily owing to moisture removal, occurring up to around 83 °C. Beyond this initial phase, the weight loss can be attributed to the dehydroxylation reactions of serpentine minerals (2Mg_3_Si_2_O_5_(OH)_4_ → 3Mg_2_SiO_4_ + SiO_2_ + 4H_2_O; refs. ^25,26^). Above 1,600 °C, haematite, which is a product from the thermal decomposition of goethite (2FeOOH → Fe_2_O_3_ + H_2_O), also thermally decomposes into a mixture of magnetite (Fe_2_O_3_) and wüstite (FeO).

**Extended Data Fig. 2:**
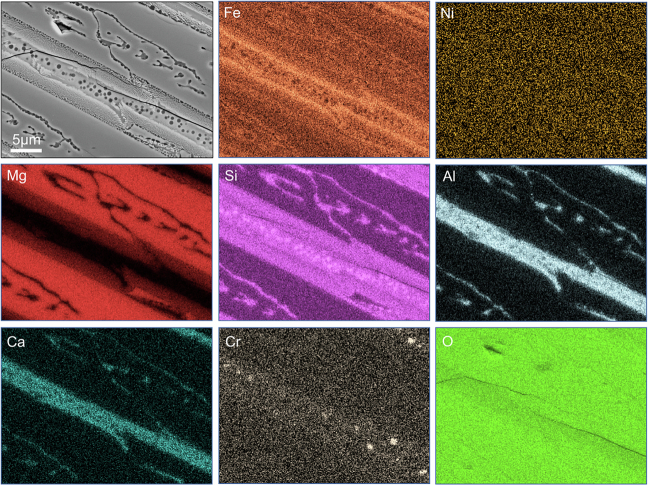
Microstructure of Ni-ore after 2 min processing in inert atmosphere (100%Ar). Microstructure and elemental mapping of Ni-ore melted in an argon atmosphere, as determined by SEM-EDX. It is evident that Ni does not selectively partition into any specific phase, exhibiting uniform dissolution across all phases. Scale bar: 5 µm.

XRD analysis of the solidified Ar-melted sample (powdered) reveals solidification primarily into olivine Mg_0.8_Fe_0.2_SiO_4_ (86.6 wt%) and pyroxene MgSiO_3_ (9.4 wt%) structure types (Fig. 2a). The absence of Ni-rich phases in the solidified sample indicates that Ni is uniformly dissolved in these silicate constituents, which was further confirmed by scanning electron microscopy and energy-dispersive X-ray spectroscopy elemental mapping, as depicted in Extended Data Fig. 2.

## Phase transformation and reduction mechanisms

**Fig. 3:**
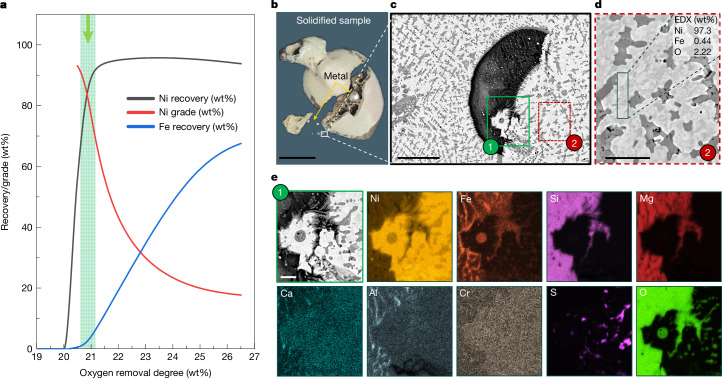
Evidence of optimal thermodynamic compositional interval for high-grade ferronickel production, a solidified sample and microstructure of the metallic droplet. **a**, Thermodynamic calculation illustrating the variation in ‘Ni/Fe recovery‘ (namely, the ratio between the Ni (or Fe) in the alloy obtained and the total Ni (or Fe) available in the original ore) and ‘Grade’ (namely, the amount of Ni (wt%) present in the Fe–Ni binary alloy) with reduction degree. The green dashed lines and the green arrow denote a thermodynamic compositional interval for tapping of highly enriched Ni alloy with optimal Ni-recovery ratio (defined as the ratio between the Ni in the alloy obtained and the total Ni available in the original ore). **b**, Solidified sample (after 2 min processing) showing the presence of an approximately 5-mm-sized metallic nodule and other smaller metal droplets solidified within the sample. **c**, Scanning electron microscopy micrograph of a small metallic droplet solidified at the bottom of the sample (depicted by the white frame in **b**). **d**, Local chemical composition of the metallic phase highlighted by the red frame in **c**, probed via energy-dispersive X-ray spectroscopy (EDX). **e**, Scanning electron microscopy micrograph and the elemental mapping of the area highlighted by the green rectangle in **c**, showing the presence of a Ni-rich metallic phase. Scale bars, 1 cm (**b**), 25 µm (**c**), 10 µm (**d**) and 6 µm (**e**).

The reduction of laterite Ni ore was performed under a H_2_-lean hydrogen-plasma arc (Ar–10%H_2_) ignited at 200 A (details in Methods). The reduction kinetics were studied by exposing the sample to Ar–10%H_2_ plasma for 2 min and 4 min. Even a brief 2-min exposure resulted in the precipitation of pure metal droplets. Rapid cooling from the water-cooled copper (Cu) hearth solidified these droplets into approximately 5-mm nodules within the unreduced silicates (Fig. 2d) or smaller 1–3 mm particles near the sample bottom (Fig. 3b). The majority of the metal was concentrated at the bottom centre of the solidified sample, suggesting complete mass separation between the silicate and metallic melts, driven by their differing viscosities and mass densities (a factor of two to three). The metallic nodules and unreduced silicates were mechanically separated after solidification. The silicate portions were weighed, powdered and analysed by XRD to track phase transformations (Fig. 2a). The processed samples (2 min and 4 min) mainly consisted of silicates, with forsterite (Mg_2_SiO_4_; about 86 wt%) and pyroxenes (MgSiO_3_; about 10 wt%). This indicates that the Ni supply comes from these silicate variants rather than a specific Ni-enriched phase. The iron-fcc (face-centred cubic) phase (Fig. 2a) detected by XRD corresponds to metal or alloy trapped within silicates that could not be mechanically separated. These minute metallic values were considered losses and excluded from subsequent calculations.

**Extended Data Fig. 3:**
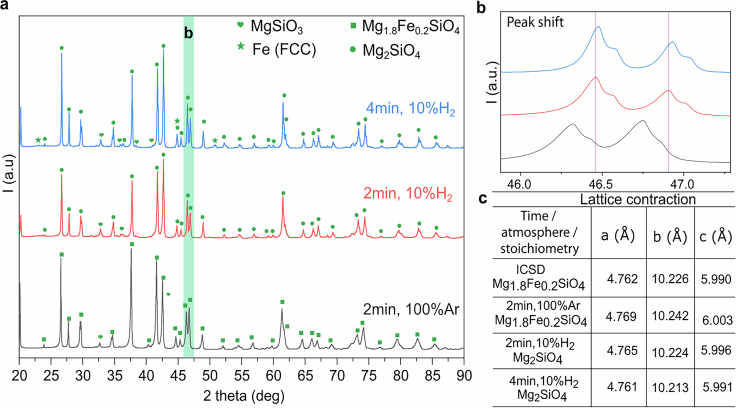
XRD data analysis of 2 min Ar-melted, 2 min & 4 min (Ar-10%H_2_) reduced Ni-ore samples, peak shifts and lattice contraction. **(a)** Comparative display the XRD patterns of samples; 100% Ar-melted for 2 min, 2 min and 4 min reduced in Ar-10%H_2_. The patterns are predominantly dominated by Mg-silicate peaks (Mg_2_SiO_4_ & MgSiO_3_), constituting over 95 wt.% of the samples. For simplification purposes, smaller peaks corresponding to Ca-Al-Fe oxides/silicates are not highlighted. **(b)** Provides an enlarged view of two peaks framed in green in (a). It is evident that these peaks shift towards the right as we progress to more reduced samples. Although arbitrarily chosen, a similar shift is observed for all peaks corresponding to the forsterite structure. **(c)** Illustrates evidence of lattice contraction in the forsterite structure as we transition from the Ar-melted sample to the 4-min sample reduced in H_2_ plasma. Additionally, it can be observed that the crystal structure of the refined Mg_1.8_Fe_0.2_SiO_4_ structure in the Ar-melted sample is expanded compared to its ICSD crystal structure. This expansion suggests that Mg ions are replaced by Ni/Fe in amounts exceeding the stoichiometric proportion indicated in the chemical formula.

The dissolution of Ni and Fe in Mg-silicate structures has been reported in the literature^23,24^. The replacement of Mg^2+^ ions (with an ionic radius of 0.65 Å (ref. ^25^)) by Ni^2+^ and Fe^2+^ with slightly greater ionic radii (0.72 Å and 0.83 Å for Ni and Fe, respectively^27^) led to expansion of the unit cell depending on their ionic concentration when embedded in the crystal structure. As an example, an expansion of 0.14%, 0.16% and 0.21% along the crystallographic *x*, *y* and *z* axis, respectively, was observed for the orthorhombic crystal structure of forsterite Mg_0.8_Fe_0.2_SiO_4_, whose cationic positions in the lattice were occupied by Fe/Ni in amounts exceeding those suggested by the stoichiometry. In Extended Data Fig. 3a,b, a comparative XRD analysis of 2-min Ar-melted, 2-min Ar–10%H_2_-reduced and 4 -min Ar–10%H_2_-reduced Ni-ore samples reveals similar patterns (Fig. 3a) with continuous peak shifts towards higher 2*θ* values (where *θ* (Bragg angle) is the angle between the incident X-ray beam and the crystal plane in XRD) (Fig. 3b). This shift signifies a lattice contraction effect owing to the progressive removal of Ni^2+^/Fe^2+^ ions from the forsterite crystal structure during reduction (Extended Data Fig. 3c). Notably, the crystal structure of refined Mg_1.8_Fe_0.2_SiO_4_ in the Ar-melted sample shows expansion compared with its ICSD (Inorganic Crystal Structure Database) counterpart (Extended Data Fig. 3c), indicating the substitution of Mg ions by Ni/Fe in amounts surpassing the stoichiometric proportions suggested in the chemical formula.

**Extended Data Fig. 4:**
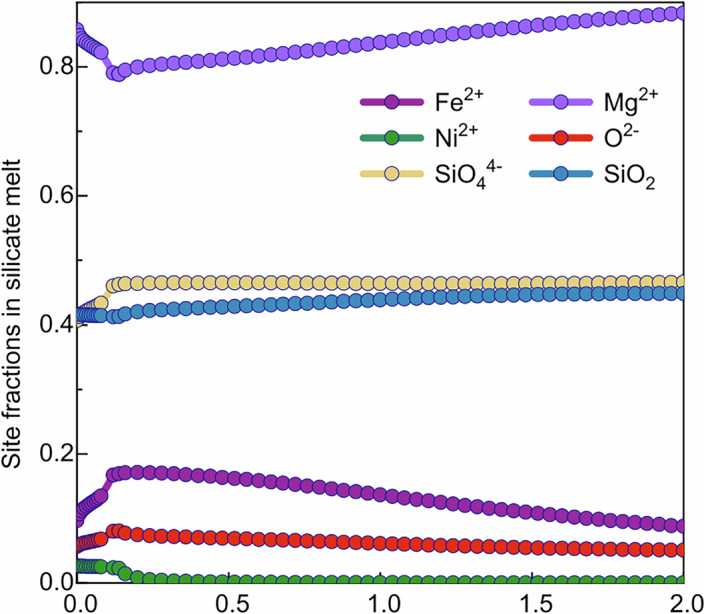
Chemical evolution of Ni-ore 1 silicate melt upon exposure to hydrogen. The figure illustrates the site fractions of various ionic species within molten Ni-Ore. Suggesting the transformation of the complex Ni-Ore system into simple system containing ionic species like Fe^2+^, Ni^2+^, Mg^2+^, SiO_4_^−4^, O^2−^, and SiO_2._ It also depicts the chemical transformation of this melt composition upon exposure to hydrogen, demonstrating the elimination of free oxygen (O^2−^). Consequently, there is a discernible selective precipitation of Ni over Fe, as evidenced by the observed decline in the site fractions of Ni^2+^ to zero at the onset of exposure.

A thermodynamic assessment (Methods) of the system’s constituent stability (Extended Data Table 2) under increasing hydrogen exposure reveals that above 1,600 °C, olivine dissociates upon melting into ionic components such as Fe^2+^, Ni^2+^, Mg^2+^, SiO_4_^4−^, O^2−^ and SiO_2_ (Extended Data Fig. 4). Oxygen in the melt exists as free ions (O^2−^), bridging oxygens (Si–O–Si, bonded to two network cations, as in SiO_2_), and non-bridging oxygens (Si–O^−^, bonded to one network cation, as in SiO_4_^4−^)^27,28^. Owing to their covalent bonds, bridging and non-bridging oxygens are more stable than free O^2−^, making them less reactive with hydrogen species^27,29^. When the ionic melt is exposed to the hydrogen plasma, chemical redox reactions occur at the arc–melt interface. Driven by chemical potential gradients, free oxygen (O^2−^) diffuses towards the interface and reacts with hydrogen-plasma species (H, H^+^ and so on), exiting as water vapour or hydroxyl radicals (OH^−^), depending on the temperature. This reaction leaves behind two electrons (2*e*^−^; Fig. 2c), which are preferentially consumed by metal cations (M^2+^) with the lowest oxygen affinity, leading to metal precipitation (M^2+^ + 2*e*^−^ → M). In the investigated system, Ni^2+^, which has the lowest oxygen affinity, precipitates first, followed by Fe^2+^. The thermodynamic assessment (Extended Data Fig. 4) illustrates the chemical evolution of the silicate melt under varying hydrogen exposure levels. Hydrogen reduces free oxygen (O^2−^) in the melt, initially depleting Ni^2+^ site fractions to zero, followed by a decline in Fe^2+^ site fractions. Site fraction definitions are provided in Methods. This thermodynamic behaviour confirms the sequential precipitation of Ni and Fe, governed by oxygen removal. Once precipitated, Ni and Fe form a homogeneous molten phase, solidifying into an Fe–Ni alloy whose composition depends on the extent of oxygen extraction, as detailed in the following section.

## Reduction pathway

Thermodynamic investigations (Extended Data Fig. 4) revealed the mechanisms governing the sequential precipitation of Ni and Fe, demonstrating the feasibility of selectively reducing Ni over Fe to obtain a high-grade Ni alloy under controlled conditions. To explore this, equilibrium calculations were conducted using ThermoCalc software, simulating the exposure of molten laterite ore (1,600 °C) to varying amounts of an Ar–10%H_2_ atmosphere. The molten pool temperature is non-uniform, with the highest values at the reaction interface (1,800–2,000 °C) and lower temperatures near the water-cooled Cu hearth^30^. Previous studies show strong agreement between thermodynamic simulations at 1,850 °C (refs. ^31,32^) and 2,000 °C (ref. ^33^) with experimental data. Given this gradient, the overall temperature probably exceeds 1,600–1,700 °C. Although higher temperatures enhance reduction kinetics, the melt pool composition evolves according to equilibrium conditions, making ThermoCalc simulations of the (Ni–Fe)-silicate–H_2_ interaction sufficient to capture compositional changes with oxygen removal. The results are shown in Fig. 3a, where Ni and Fe recovery from the molten ore and the corresponding Ni–Fe alloy composition (that is, alloy grade) are plotted against the oxygen removal degree. The oxygen removal degree is calculated from the oxygen mass loss owing to either thermal decomposition (see differential scanning calorimetry plots in Extended Data Fig. 4) or its reaction with hydrogen (O^2−^ + H_2_ → H_2_O + 2*e*^−^). Recovery is defined as the ratio of Ni (or Fe) in the alloy obtained to the total Ni (or Fe) content in the original ore. This includes Ni/Fe present in the obtained alloy to the total Ni (or Fe) in the original ore, excluding fine metallic droplets lost to slag during slag–metal separation.

Figure 3a shows that metal precipitation begins only after oxygen removal reaches about 20%. The associated oxygen loss is due to dehydroxylation and thermal transformations at high temperatures and low partial pressures (see ‘Original laterite Ni ore’). Notably, this 20% oxygen removal occurs without hydrogen consumption, as weight loss results solely from thermal processes. To trigger metal precipitation, further oxygen removal is required, necessitating hydrogen exposure.

The green dashed lines and arrow in Fig. 3a indicate that there exists a thermodynamic compositional interval, spanning a reduction degree of 20.7% to 21.3%, with the potential to yield high-grade ferronickel (that is, with 70–90 wt% Ni content) with corresponding total recovery rates ranging from 60 wt% to 90 wt%. To utilize this thermodynamic compositional interval effectively, it is essential to introduce a stoichiometric amount of hydrogen into the system, aligning with an oxygen removal degree in the range of 20.7% to 21.3%.

To validate the thermodynamic assessment shown in Fig. 3a, we focused on the smallest alloy particles obtained after 2 min of hydrogen-plasma exposure. Although the plasma arc’s direct interaction with the melt pool induces strong stirring that promotes coalescence of metallic droplets, some droplets may escape the turbulent flow and solidify independently before coalescing into larger droplets. These smaller droplets, which solidify before coalescing, can serve as a micro-lab, representing instantaneous metal precipitation events. Some of these particles may originate from the early stages of reduction and are expected to exhibit high Ni content, as indicated by the white frame in Fig. 3b.

The microstructure of the particle in Fig. 3c primarily consists of a bright metallic phase and micrometre-sized dendrites of unreduced Ni-rich oxides. Energy-dispersive spectroscopy analysis (Fig. 3d) reveals a high Ni content of 97.3 wt%, with minor Fe (0.44 wt%) and O (2.22 wt%). This composition aligns with the calculated selective thermodynamic compositional range (Fig. 3a), supporting the feasibility of selective Ni reduction over Fe, as predicted by thermodynamic theory. In addition, an entrapped Mg-silicate slag droplet was observed within the metallic particle, caused by the strong melt turbulence induced by the arc, as shown in Fig. 3c. The slag–metal interface and corresponding elemental mapping are shown in Fig. 3e.

**Extended Data Fig. 5:**
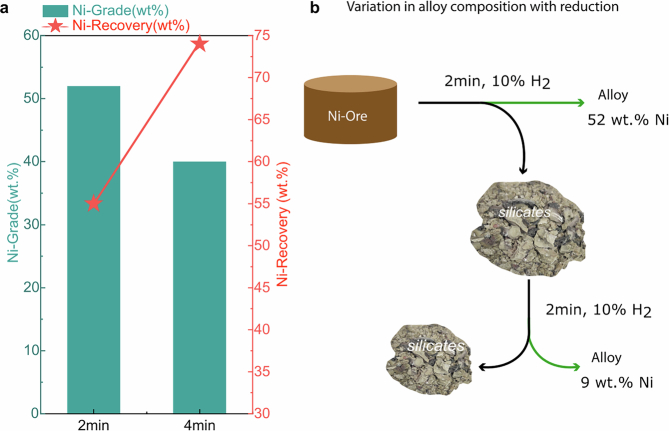
Tracking Ni-Grade (amount of Ni (wt.%) present in the Fe-Ni binary alloy)/Recovery (amount (wt.%) of Ni recovered in the form of alloy from the original ore) over time. **(a)** Shows the variation of Ni-grade (Ni-content (wt.%)) (teal bars) and recovery (amount (wt.%) of Ni recovered in the form of alloy from the original ore) (red stars) of metallic nodules obtained after 2 min and 4 min processing in lean hydrogen plasma (Ar-10%H_2_). **(b)** Variation in alloy composition with reduction progression: The ore was reduced for 2 min in Ar-10%H_2_ and the corresponding metal and silicate portions were mechanically separated and the letter was further subjected to reduction with a plasma arc ignited under the identical conditions, the Ni-content in the later alloy was found to be lower (9 wt.%) than the former alloy (52 wt.%).

Extended Data Fig. 5a shows the Ni grade and recovery degrees for samples reduced in Ar–10%H_2_ for 2 min and 4 min. After 2 min, 55 wt% of Ni was extracted as metallic nodules, mechanically separated from the slag, and confirmed by scanning electron microscopy and energy-dispersive X-ray spectroscopy to contain 52 wt% Ni. After 4 min, the Ni grade reached 42 wt%, with recovery increasing to 74 wt%.

The decline in the Ni grade (amount of Ni (wt%) in the alloy) after 4 min indicates that continued reduction leads to Fe enrichment and Ni depletion in the alloy, as shown in Fig. 3a. This was confirmed by reprocessing the unreduced silicate from the 2-min plasma-processed sample for another 2 min under Ar–10%H_2_ plasma (Extended Data Fig. 5b), yielding an alloy with 9 wt% Ni and 90 wt% Fe.

## Process flexibility and atmosphere control

**Fig. 4:**
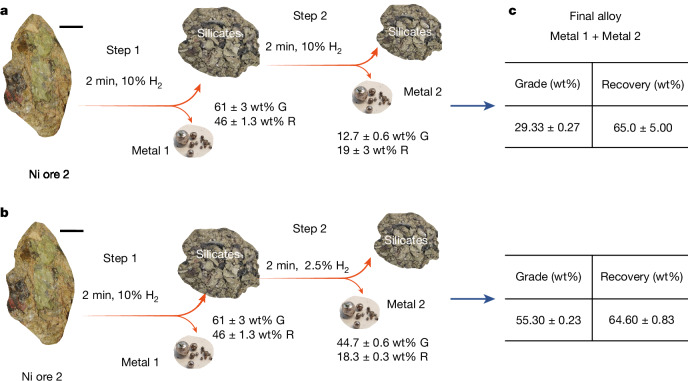
Tracking both Ni grade present in the Fe–Ni binary product alloy and recovery degree. **a**,**b**, Experimental protocol for a two-step experiment designed here to emphasize the significance of proper atmosphere control over the reduction time. In **a**, the ore was reduced for 2 min in step 1. The corresponding metal and silicate portions were mechanically separated and the silicate was then subjected to further reduction using a plasma arc ignited with an atmosphere of Ar–10%H_2_. This second reduction sequence was denoted as step 2. In **b**, step 2 was conducted with a mixture of only Ar–2.5%H_2_, to make the process as H_2_-lean as possible and increase the Ni content extracted. G, Ni grade (amount of Ni in wt%); R, Ni recovery (amount, wt%), which refers to the amount of Ni extracted from the original ore. **c**, Comparison of the weighted averages of the grade and recovery for the alloy (metal 1 + metal 2) obtained in steps 1 and 2 of the experimental protocols described in **a** and **b**. Scale bars, 1 cm.

To assess the flexibility of HPSR in processing different Ni ores, a second type of Ni laterite ore containing slightly more Ni (1.57 wt%) and Fe (16 wt%) was also studied. This ore is hereafter referred to as ‘Ni ore 2’. The detailed chemical and phase composition of Ni ore 2 is documented in the Extended Data Tables 3 and  4, respectively. This ore variant was processed under similar conditions to the ones adopted for the first Ni ore studied here, as described in Methods. A 2-min reduction process leads to the formation of high-grade (65 wt% Ni) metallic nodules, translating to a recovery of 45 wt% Ni (denoted as ‘step 1’ in Fig. 4a). Further processing of the unreduced silicates that had been obtained after 2 min for an additional 2 min under identical conditions (Ar–10%H_2_) resulted in the formation of a second portion of metallic nodules with a grade of only 12.4 wt% Ni (‘step 2’ in Fig. 4a). By combining the results from these two steps, as described in Fig. 4a,c, the overall grade (Ni content (wt%)) of all metallic droplets obtained after a cumulative processing duration of (2 + 2) min was about 30 wt% Ni, which translates to a global Ni recovery of 65 wt%. This result suggests that our procedure could be applied to a wider range of Ni-containing silicates. However, prolonged exposure to hydrogen-containing plasma reduces the ferronickel grade, as shown in Extended Data Fig. 5. These findings highlight the importance of consistently monitoring and controlling the reducing atmosphere to optimize the Ni concentration in the final alloy.

In this sense and to elucidate the impact of the H_2_-containing atmosphere control on the Ni content of the recovered metallic nodules, we adopted the two-step experimental protocol depicted in Fig. 4b. This approach involved processing the Ni ore 2 in two steps with different plasma atmospheres: in step 1, the ore was exposed to a 2-min treatment in Ar–10%H_2_, followed by separation of the metallic nodules from the unreduced silicates. In step 2, the unreduced silicates (obtained in step 1) were subjected to an additional 2-min processing in Ar–2.5%H_2_. The resulting alloy obtained after exposure to the Ar–2.5% H_2_ plasma (step 2, Fig. 4b) resulted in a significant enrichment in Ni, with a content of about 45 wt%. By considering the metal portions obtained in both steps 1 and 2, the overall grade of the alloy is about 55 wt% Ni, a value that can be translated into a recovery of about 64 wt% of the total Ni present in the original ore (Fig. 4b,c). Comparing the results of this atmosphere-controlled experiment (Fig. 4a,c) to the one processed without any control (Fig. 4b,c), we observe that after only an effective 4-min processing duration, a higher-grade product is obtained (in controlled atmosphere) without compromising the degree of metal recovery.

The two-step reduction process shown in Fig. 4 is meant to illustrate the effect of the reducing atmosphere on nickel grade and recovery. In practice, this process can run continuously, similar to conventional EAF operations, without the need for stopping the process for gas adjustments. The remaining slag can be further processed in the continuous cycle to recover the remaining Ni. The silicates obtained after 4 min (step 2) of the reduction were further processed in Ar–2.5%H_2_ for 4 min, which allowed to tap a ferronickel of grade 10 wt% Ni, which translates to the overall recovery of 78 wt% Ni. Combining the alloys obtained from each step, we get an alloy containing 30 wt% Ni.

**Extended Data Fig. 6:**
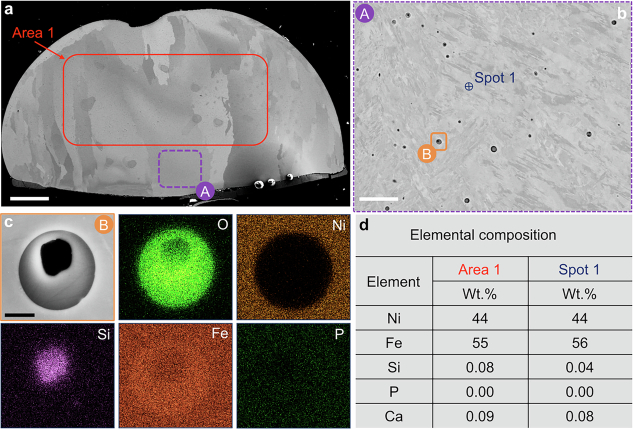
Microstructure and composition of the metallic phase after 4 minutes of processing. **(a)** Microstructure of the metallic nodules. **(b)** Enlarged view of the metallic region (highlighted in pink, A) showing micron-sized inclusions within the metal. **(c)** Elemental mapping of an inclusion (highlighted in orange, B), showing a Si-rich core surrounded by Fe oxides. **(d)** Overall composition of the region highlighted in red (Area 1) shows 44 wt.% Ni, with minimal impurities (Si ~0.08 wt.%, P ~ 0.00 wt.%, Ca ~0.09 wt.%). Spot analysis of an inclusion-free region (spot 1, (b)) reveals a dissolved Si content of less than 0.04 wt.%, significantly lower than typical commercial ferronickel alloys (Si up to 0.3 wt.%). Scale bar: (a) 300 µm, (b) 5 µm, (c) 1 µm.

Extended Data Fig. 6 shows the microstructure and composition of the metallic phase obtained after 4 min of processing (from step 2, Fig. 4b), as determined by scanning electron microscopy and energy-dispersive X-ray spectroscopy. The overall composition of the area highlighted in red (area 1) is 44 wt% Ni, with minimal impurities, including Si (about 0.08 wt.%), phosphorus (P; about 0.00 wt%) and Ca (about 0.09 wt%).

## Discussion

**Fig. 5:**
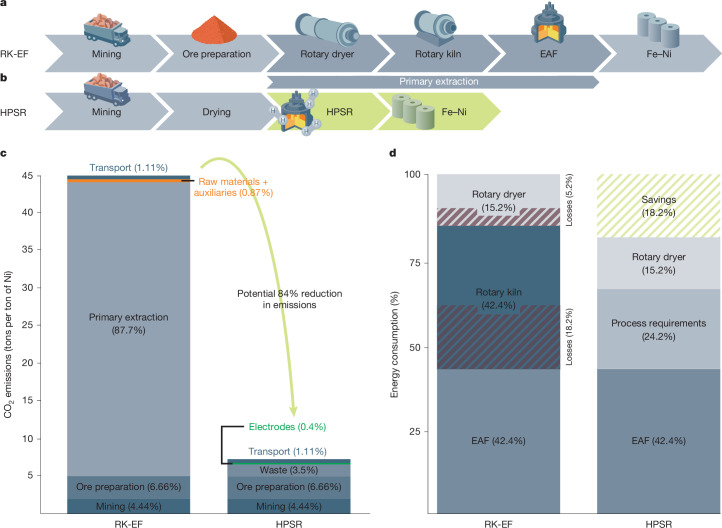
Comparative analysis of CO_2_ emissions and energy efficiency in traditional RK-EF route and HPSR alternative. **a**, Different steps involved in the traditional RK-EF route. **b**, HPSR as a one-step processing alternative to RK-EF. **c**, Comparison of CO_2_ emissions from the traditional RK-EF route and HPSR. Data from ref. ^24^. The emissions from mining, ore preparation (crushing, screening and partial moisture removal), waste disposal, electrode consumption and transport are assumed to remain the same in both cases. **d**, Comparison of input energy consumption in different processing steps between the RK-EF and HPSR routes. Data from ref. ^23^. The illustration highlights that energy used in rotary dryers and rotary kilns in RK-EF is not fully utilized for the process requirements, resulting in approximately 5.2% and 18.2% losses to the environment, respectively (represented by maroon strips). Direct dried ore charge processing in the HPSR route can mitigate these losses, achieving energy savings of up to 19% (depicted by light green strips in the HPSR column).

We have demonstrated the feasibility of extracting metallic Ni from highly diluted ore without carbon-based fuels or reductants. Next, we present a theoretical evaluation to understand the thermodynamic foundations of this process, enabling knowledge-based protocols for industrial upscaling. Traditional Ni laterite processing (RK-EF) is a multistep, energy-intensive route involving: (1) ore drying in a rotary dryer, (2) calcination and partial reduction in a rotary kiln, and (3) smelting in an EAF^34^, relying on carbon-based fuels (bituminous coal, natural gas). Figure 5a details the RK-EF process and its CO_2_ emissions, compared with the proposed HPSR process in Fig. 5b. Figure 5c breaks down the CO_2_ contributions per processing step: mining and ore preparation contribute only about 4.4% and 6.6%, respectively, whereas the primary extraction step, involving emissions from the rotary dryer, rotary kiln and EAF operations, accounts for a significant 87% of the overall CO_2_ emissions^34^. In contrast to this lengthy processing route, our one-step HPSR process (Fig. 5b) bypasses the pre and post treatments like calcination and pre-reduction of ore in rotary kilns, refining of the crude FeNi in ladle furnaces or converters. The entire dried ore charge is processed directly in an EAF without any preceding refinement, using renewable electricity and a minimal amount of sustainably produced hydrogen as a green reductant. Preliminary calculations (Supplementary Information section ‘CO_2_ emissions and energy assessments’) estimate an 84% reduction in CO_2_ emissions with this method, as shown in Fig. 5c, assuming full reliance on renewable energy, a highly plausible scenario in the near future with the ongoing green energy transition.

The energy required for the pre-treatments accounts for about 57% of the total energy required for the entire RK-EF process, with rotary dryers consuming about 15% and rotary kilns about 42% (ref. ^35^; Fig. 5d). Among the total energy consumed in these steps only about 27% of the energy is required to thermally run and maintain the process, such as heating of the ore, decomposition reactions of serpentine minerals, removal of free moisture and chemically bonded water, and the chemical redox reactions^35^. From the rest, about 7% of the energy is reutilized as a heating source^35^ and the remaining fraction is lost to the environment in the form of flue gas losses, thermal losses to air during the transfer of materials and dust losses, to mention a few of the main parameters. As an example, the calcined ores leave the rotary kiln at an average temperature of 880 °C and enter the EAF at about 400 °C (ref. ^35^). The energy loss during this transfer step alone in today’s production practice accounts for about 8% of the total energy consumed along the entire extraction process^35^.

By bypassing all these energy- and CO_2_-intensive pre-treatments and instead processing the entire dried ore charge in an EAF in a single smelting reduction step, all these losses can be avoided to make the process energy efficient. On the basis of the calculation, as shown in Supplementary Information section ‘CO_2_ emissions and energy assessments’, it is estimated that energy savings of up to 18% can be achieved by using HPSR to produce Fe–Ni from laterite ores, as also shown in Fig. 5d. Ore drying can be efficiently achieved by utilizing the outflow gas mixture of H_2_ and H_2_O. Hot steam can directly heat the drying vessel (for example, a rotary dryer) and unutilized H_2_ can be combusted for additional heat. Any remaining H_2_ can be recirculated to the plasma furnace after dust filtration and water condensation, a process that is well established in hydrogen-based direct-reduction iron reactors^36^. Furthermore, the alloy obtained during the process is highly enriched in Ni (about 30%) and it can be utilized directly for stainless steel, invar or magnet alloy making without any energy-intensive refining treatments (Fig. 5b).

When it comes to the technical perspective for using this at larger scale, one of the crucial aspects for upscaling this technology involves ensuring adequate mass transport of the unreduced melt to the reaction interface. This is significant because most of the chemical reactions during the process occur at the arc–melt interface, with free oxygen (O^2−^) consistently being removed by hydrogen-plasma species. This means that for the reaction to advance, a continuous supply of free oxygen species (O^2−^) must be maintained at such an interface. This continuous supply is sustained by strong melt stirring, driven by temperature gradients within the volume^31^ and electromagnetic forces from the electric current in d.c. mode^37^. In large-scale systems, mass transfer during smelting can be enhanced by using short arcs with high currents and increasing the number of electrodes in a.c. EAFs^37,38^. Alternatively, external electromagnetic stirring^39^ or bottom gas injection^40^ can create localized vortices, significantly improving melt stirring. These technologies are already well established in the industry. In addition, maintaining low viscosity is crucial for efficient mass transport. This occurs naturally as complex ore constituents such as lizardite, pyrope, goethite, pimelite and silica (Fig. 2a) transform into a simpler Mg-silicate system (Fig. 2a). The conversion of polymeric SiO_2_ into SiO_4_^4−^ monomers further reduces viscosity, facilitating melt flow^41^.

All in all, HPSR allows the extraction of a high-grade Ni-enriched Fe–Ni alloy in a single metallurgical step from dried ore feedstock, suitable for the stainless-steel or special metals industry. Alternatively, the alloy can undergo further refinement (for example, matte smelting or hydrometallurgical treatments) for specialized applications such as battery electrodes. The process produces Mg-silicate slags, which can be used in brick, road and cement production or refined for use in the cosmetic industry^42^. This study demonstrates HPSR as a sustainable and energy-efficient method for Ni extraction from low-grade laterite ores. Thermodynamic control of the reduction atmosphere optimizes Ni reduction, directly forming a high-grade Fe–Ni alloy with low impurities (<0.04 wt% Si, about 0.01 wt% P and <0.09 wt% Ca), eliminating the need for further refining. Powered by renewable energy and using green hydrogen, the process could potentially achieve 18% energy savings and reduce CO_2_ emissions by up to 84%. Furthermore, a detailed cost analysis (detailed in Supplementary Information section ‘Cost analysis’) highlights the positive financial outcome, reinforcing the possible commercial viability of this sustainable alternative to conventional Ni production.

## Methods

### Material

The material used in this research comprises Ni ore, specifically of saprolite type, directly sourced from the mine. The as-received ore manifested as rocks of varying sizes (refer to Fig. 2b). To achieve a consistent composition, the ore underwent ball milling to achieve an approximate size of 100 µm. The chemical and phase composition of the ore was determined using inductive coupled plasma optical emission spectroscopy and XRD, respectively, and is documented in Extended Data Table 1. For the validation experiments, a distinct type of saprolitic ore was utilized, and its chemical and phase composition is detailed in Extended Data Table 2.

### Thermogravimetric analysis–differential scanning calorimetry

To identify mass changes and energy endo- and exothermic reactions, associated with phase transitions, thermogravimetric analysis coupled with differential scanning calorimetry experiments were carried out in an SDT Q600 thermal analyser, using a heating rate of 20.00 °C min^−1^ and an Ar flow of 100.0 ml min^−1^.

### Reduction experiments

The Ni-ore fines were compacted into green pellets under a maximum pressure of 20 × 10^6^ Pa. Approximately 10 g of the pellets was placed on the water-cooled Cu hearth of a conventional arc melting furnace equipped with a tungsten electrode (6 mm in diameter). The furnace chamber (18 l) was purged with a gas mixture of (Ar–10%H_2_ or Ar–2.5%H_2_) at a total pressure of 900 mbar. An arc plasma was initiated between the electrode and the input material at 200 A. Simultaneous melting and reduction occurred during a 2-min exposure to hydrogen plasma. Subsequently, the arc turned off, the sample solidified and the chamber atmosphere was replenished with a fresh gas mixture. This procedure was repeated once for the 2-min processed sample and twice for the 4-min processed sample.

### Phase quantification

Small sections were cut from the solidified samples for microstructural analysis. The remaining portion was subjected to hammering to separate metallic nodules of millimetre size from the remaining silicates. The corresponding silicate portions were further pulverized and analysed via XRD. XRD was performed in a Bragg-Brentano configuration using a Cu Kα source. The 2*θ* scan range was set from 20° to 120° with a sampling step of 0.01°. The XRD data were analysed through Rietveld refinement. XRD facilitated the quantification of micrometre-sized metallic domains entrapped within the silicate portions and the determination of lattice constants for the identified phases.

### Microstructural characterization

Representative samples underwent metallographic preparation through grinding, polishing and finalization with silica particle OPS (oxide polishing suspension) polishing. Metallographic investigation was conducted using a Zeiss Merlin microscope via high-resolution scanning electron microscopy, which included energy-dispersive X-ray spectroscopy.

### Thermodynamic calculations

Equilibrium calculations were conducted using ThermoCalc software in conjunction with the TCS Metal Oxide Solutions Database (TCOX10) to describe the liquid phases and the SSUB5 SGTE (Scientific Group Thermodata Europe) Substances Database for characterizing the gas phase, inclusive of metallic and oxide vapours.

The liquid phase was described by a two sublattice model considering the following species: (Fe^+2^_,_ Mg^+2^, Ni^+2^_,_ Si^+4^)_1_(O^−2^, SiO_4_^−4^, VA^−^, FeO_1.5_, SiO_2_)_1_, where charged vacancies vary with the composition to keep electro-neutrality. The position occupied by each of these species in the sublattice is called ‘site fraction’.

To establish boundary conditions, a 10 g mass of Ni ore with the chemical composition outlined in Extended Data Table 1 was utilized. The molten Ni ore was maintained at 1,600 °C while progressively introducing varying quantities of a gas mixture containing Ar–10%H_2_. During the simulations, element partitioning was allowed among all constituents. The total pressure was maintained at 0.9 × 10^5^ Pa, the same as that used for experiments.

### CO_2_ emissions and energy assessments

The CO_2_ emissions were calculated using extracted values from ref. ^24^, and the energy comparisons with traditional RK-EF routes were calculated based on the data in ref. ^23^. Detailed descriptions of the calculations are provided in full in Supplementary Information section ‘CO_2_ emissions and energy assessments’.

## Online content

Any methods, additional references, Nature Portfolio reporting summaries, source data, extended data, supplementary information, acknowledgements, peer review information; details of author contributions and competing interests; and statements of data and code availability are available at 10.1038/s41586-025-08901-7.

## Supplementary information

Supplementary Information

## Figures and Tables

**Extended Data Table 1:**
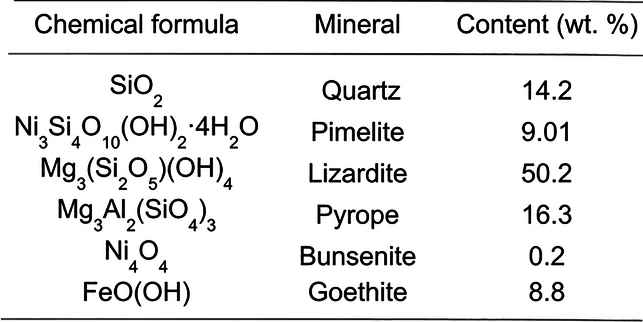
Phase composition of as -received Ni-ore 1

Phase constitution of the as-received Ni-ore determined by XRD.

**Extended Data Table 2:**
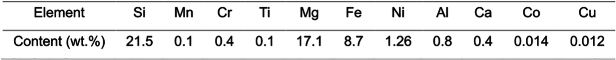
Elemental composition of as-received Ni-ore 1

As-received Ni-ore elemental composition as determined by MP-AES (microwave plasma-atomic absorption spectroscopy).

**Extended Data Table 3:**
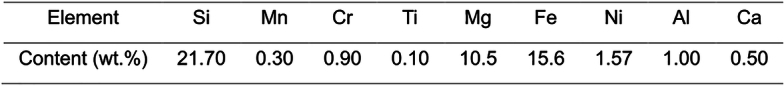
Elemental composition of as-received Ni-ore 2 used for validation experiments

As-received Ni-ore 2 elemental composition as determined by ICP-OES (inductive coupled plasma-optical emission spectroscopy).

**Extended Data Table 4:**
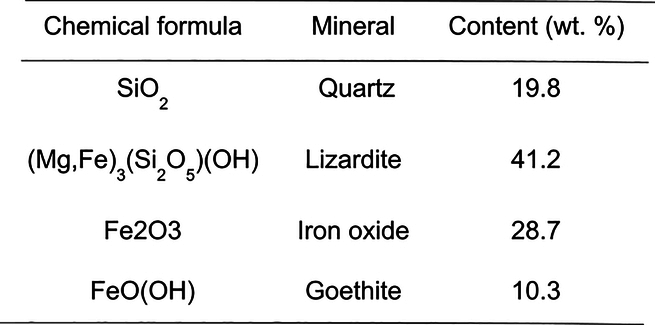
Phase composition of as -received Ni-ore 2 used for validation experiments

Phase composition of as-received Ni-Ore determined by XRD.

## Data Availability

The data are available from the corresponding author.
