# Supplementary material for: Sustainable nickel enabled by hydrogen-based reduction
Source: Nature. 2025 Apr 30;641(8062):365–73. doi: 10.1038/s41586-025-08901-7 (PMC12058512; doi:10.1038/s41586-025-08901-7)
Supplement: Supplementary file 1 — Supplementary Information [file 41586_2025_8901_MOESM1_ESM.pdf]

nature portfolio

https://doi.org/10.1038/s41586-025-08901-7

Supplementary information

Sustainable nickel enabled by hydrogen-based reduction

In the format provided by the authors and unedited

Sustainable nickel enabled by hydrogen-based reduction

U. Manzoor[1], L. Mujica Roncery[2], D. Raabe[1], I. R. Souza Filho[1,3,*]

[1]Max-Planck-Institut for Sustainable Materials GmbH, 40237 Düsseldorf, Germany
[2]Universidad Pedagógica y Tecnológica de Colombia, Tunja 150003, Boyacá, Colombia
[3]Institut Jean Lamour, CNRS (UMR 7198), Université de Lorraine, F-54000, Nancy, France

*Corresponding authors: i.souza@mpie.de

Industrial processing routes of Ni-laterites.

HPAL

High-Pressure Acid Leaching (HPAL) is a hydrometallurgical extraction technique employed in the processing of Ni and Co (if present) from lateritic ores, specifically for limonite ores containing low MgO content[43]. The process entails slurrying the ore and heating it at high temperatures (250-225°C) and pressures (30-60 bar) in a titanium-clad autoclave with steam and sulfuric acid (H_2SO_4), resulting in the leaching of Ni into an acidic solution, which is then separated and further processed to obtain final products such as electro-nickel, nickel oxide, or nickel briquettes. H_2SO_4 acts as a leaching agent, promoting the breakdown of mineral structures and facilitating the release of Ni ions into solution[9]. The elevated pressure and temperature enhance the reaction kinetics, allowing for efficient metal extraction. These processes consume large quantities of water and aggressive chemicals (e.g., to produce 1 kg of Ni via leaching, 660 L of water and 66 kg of sulfuric acid are required)[44], necessitating the appropriate neutralization, disposal and/or recycling of the by-products.

RK-EF/blast furnace

The rotary kiln and electric arc (or blast) furnace establish a pyrometallurgical process combination mainly utilized for saprolitic ores which contain Ni dissolved in a hardly reducible Mg-silicate structure. The route involves drying and pre-reduction of ore charges mixed with carbon-based reductants in rotary kilns at ~1000°C, followed by high temperature smelting (1600-1800 °C) in electric arc furnace or blast furnaces[10,11]. Coke also participates in the smelting step to separate the magnesia-silica-based compounds in the form of slag from the product ferronickel which can serve as a precursor for instance for stainless steel production[45,46]. During the

calcination stage, carbon monoxide (CO) serves as the reducing agent, but the reduction kinetics is relatively slow (~40% of reduction reactions are accomplished in rotary kilns). The energy required to heat the ore is provided by the combustion of carbon-based fuels, which are economically favorable compared to the electricity used in the EAF. While the reactions in the kiln are exothermic (Table S1), they proceed slowly, but the cheaper fuel offsets this limitation. In the EAF, the remaining 60% of the reduction takes place, utilizing solid carbon (coke) as the reducing agent. The corresponding reactions are highly endothermic (Table. S1), requiring substantial amounts of electricity, but the reduction kinetics is faster than that occurring in the kiln. The combination of slower, exothermic reactions in the kiln with faster, endothermic reactions in the EAF allows the process to balance energy efficiency and costs. This two-step reduction minimizes the need for costly electrical energy, making the process more economically viable.

Table. S1 Enthalpy change of the reactions occurring in Rotary kilns and EAF during RK-EF processing of Ni-Laterites, Data adapted from Ref [47].

	**Reactions**	**ΔH (kJ/mol)**
**Calcination /Reduction (800°C)**	$CO + 3Fe_2O_3 = 2Fe_3O_4 + CO_2$	-29.76
	$CO + Fe_3O_4 = 3FeO + CO_2$	16.35
	$CO + FeO = Fe + CO_2$	-8.98
	$CO + NiO = Ni + CO_2$	-20.63
**Smelting (1450°C)**	$C + 3Fe_2O_3 = 2Fe_3O_4 + CO$	125.68
	$C + Fe_3O4 = 3FeO + CO$	260.22
	$C + FeO = Fe + CO$	132.97
	$C + NiO = Ni + CO$	87.59

Pyrite (FeS), as a sulphur-carrier material, can also be charged into EF concomitantly with the pre-reduced ores (coming from the rotary kiln) in order to form Ni-based sulphide products known as matte[48-50]. Matte can be further processed via autoclave dissolution and solvent extraction to obtain battery-grade Ni. Using S in these processes leads to SO_2 emissions, a noxious and reactive gas that is responsible for causing the so-called “acid rain”.

Solid-state direct reduction of Ni-Laterites

Hydrogen is emerging as a promising green reducing agent for extracting technologically significant metals from their ores, particularly oxides. For instance, in ironmaking sector, iron ores, primarily hematite, are reduced at 850-1000°C[20,51,52] (successfully demonstrated at industrial scale) through the solid-state chemical reaction: $Fe_2O_3 + 3H_2 \rightarrow 2Fe + 3H_2O$. This process

generates water as the direct by-product, ensuring that very limited amounts of direct greenhouse gases are emitted. From the few studies about solid-state direct reduction (DR) of Ni-laterites using molecular hydrogen (H_2), the chemical reactions between H_2 molecules and the containing goethite (viz., (Ni,Fe)OOH) compete with the densification of the constituents, a fact that decreases the density of open porosity for inbound of hydrogen gas and outbound flux of product gases[21]. Under these circumstances, the reduction reaction proceeds with sluggish rates, yet with ever-inefficient consumption of hydrogen gas[18]. The final product is a solid material composed of a mixture of unreduced oxides encapsulated by layers of a ferronickel. Hence proper separation of the metallic product from unreduced oxides require post magnetic separation or chemical processes[19].

In the case of saprolites, in which Ni is dissolved in hardly reducible olivine-like constituents (M_2SiO_4, where M is a divalent cation, including Ni^{2+}), the direct reduction of these ores with gaseous hydrogen is hardly reported in literature. This might be primarily associated to the fact that olivine is thermodynamically stable at the temperature interval normally adopted in DR (800-1000°C)[18,21], and its reduction by molecular hydrogen might not be feasible without preceding efforts to chemically transform the complex minerals (e.g., Ni_2SiO_4) into simpler substances, such as NiO[21,22]. The chemical conversion of the mineral to a simplified oxide entails a thermal treatment (~1100°C) in the presence of a catalyst (e.g., Na_2SO_4) and H_2 gas. This catalytic process facilitates the replacement of Ni^{2+} ions encapsulated within magnesium-containing silicate compounds ($Ni_2Mg_2Si_2O_7$), through an ionic exchange with Na^{2+} ions derived from Na_2SO_4. Consequently, forming a low-melting eutectic phase, $Na_2Mg_2Si_2O_7$, along with Na_2SiO_3, SO_2 and NiO[22].

Definition of Ni/Fe-grade, elemental recovery and reduction degree

Ni-recovery (%) = $\frac{mass\ of\ Ni\ in\ alloy}{mass\ of\ Ni\ in\ ore} \times 100$

Ni-grade (%) $= \frac{mass\ of\ Ni\ in\ alloy}{mass\ of\ (Fe+Ni)\ in\ alloy} \times 100$

Fe-recovery (%) $= \frac{mass\ of\ Fe\ in\ alloy}{mass\ of\ Fe\ in\ ore} \times 100$

Oxygen removal degree (%) $= \frac{mass\ of\ O\ removed\ from\ sample}{total\ mass\ of\ O\ in\ ore} \times 100$

The term "recovery" refers exclusively to the Ni/Fe content recovered as metallic nuggets and does not include the negligible amounts of metallic droplets entrapped within the slag.

Definition of "single metallurgical step" process

The term "single step" in this manuscript refers to the production of refined ferronickel from dried ore feed in a single metallurgical process as compared to RKEF route. The RKEF process involves three distinct metallurgical stages. First, the dried ore is **calcined**, which involves heating the ore to temperatures of up to 1000°C in the presence of reducing agents. This step ensures complete drying and partial reduction of Fe-Ni oxides and silicates into their metallic forms. The calcined ore is then **smelted** in an electric arc furnace (EAF), where the remaining chemical reactions occur, producing a crude ferronickel alloy that contains impurities such as carbon (C), sulfur (S), silicon (Si), phosphorus (P), and calcium (Ca). Lastly, **refining** is required to reduce these impurities to acceptable levels.

In contrast, the Hydrogen Plasma Smelting Reduction (HPSR) process integrates **calcination**, **smelting**, and **refining** into a single step. The drying of the ore which involves removal of moisture from the ore can be realized by re-utilization of the outflow gasses going out of electric arc furnace. the gas mixture is predominantly rich in H_2O and H_2 at exits the furnace at high temperatures can directly be utilized for drying of the ore. The dried ore feed is directly introduced into the hydrogen plasma reactor, producing high-grade refined ferronickel. This alloy contains very low levels of impurities, with silicon (Si) below 0.08 wt.%, calcium (Ca) below 0.09 wt.%, and phosphorus (P) nearly 0.00 wt.%. The impurity levels of the ferronickel produced by HPSR are lower than those in commercially available ferronickel.

CO_2 emissions and Energy assessments

a) CO_2 emissions

The comprehensive LCA assessments of Ni laterite processing by Rotary Kiln-Electric Furnace (RK-EF) process documented in the literature reveals an average of 45 tons of CO_{2e} emitted per ton of nickel produced (ton Ni)[34]. The Ni extraction through this route primarily

consists of four stages: 1) mining of the ore, 2) ore preparation, 3) primary extraction, and 4) transport of the final product, as schematically shown in Supplementary Fig. 1

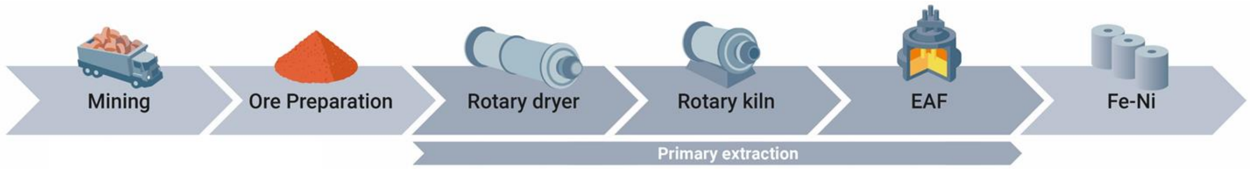

Supplementary Fig. 1. Different steps involved in the traditional Rotary Kiln Electric Arc Furnace (RK-EF) route.

Table S2 provides a breakdown of CO_2 emissions associated with each of these steps, with data adapted from ref. [34]. Notably, the primary extraction emerges as the predominant contributor, responsible for 87.7% of the total CO_2 emissions[34]. Ore preparation involves activities such as crushing, screening, and partial drying of the ore charge by utilizing energy derived from the combustion of carbon-based compounds, predominantly bituminous and coal[35]. The subsequent primary extraction phase entails the transformation of the partially dried ore into Fe-Ni alloy and slag. This step uses carbon-based substances both as reductants and energy sources, a fact that results in huge CO_2 emissions (45 tons CO_{2e}/ton Ni) and makes the Ni as one of the most harmful commodities to use (See Fig. 5c).

Table S2. CO_2 emissions from different steps involved in Ni-laterite processing through RK-EF route (data adapted from ref.[34]), also shown in Fig. 5c.

Process step	Contribution to total CO_2 emissions in RK-EF route (%)
Mining	4.44
Ore-preparation	6.66
Primary extraction	87.77
Transport	1.11

In the proposed hydrogen plasma smelting reduction route (HPSR), the entire dried ore undergoes direct processing, as shown in Supplementary Fig. 2.

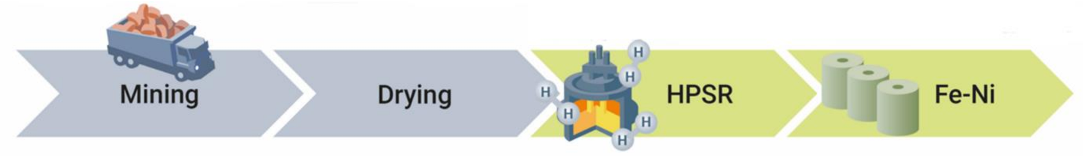

Supplementary Fig. 2. Hydrogen Plasma Smelting Reduction (HPSR) as a one-step process, wherein the entire dried ore charge is processed directly without any pre-treatments.

Hence, the possibility of reducing the CO_2 emissions in Ni production through HPSR was calculated considering the following specific factors:

1) The process is assumed to be exclusively powered by renewable electricity sources, and green hydrogen completely replaces carbon-based reductants;

2) Mining of laterite ores and the transportation of final products are assumed to employ traditional methods, thus maintaining the current emission levels from these sectors as reported in Ref. [34];

3) Following Ref. [34], we also assume that the emissions coming from waste disposal and electrode consumption in HPSR are the same as the ones found in the "primary extraction steps" of the conventional RK-EF route, highlighted in Supplementary Fig. 1.

Considering these factors, the total CO_2 emissions from HPSR process are calculated as follows:

$$\text{Total } CO_2 \text{ emissions} = \text{Mining emissions} + \text{Ore preparation} + \text{Primary extraction (i.e., emissions from electrode consumption and waste disposal emissions)} + \text{Emissions from transportation of final products}$$

From the LCA study published by the Nickel Institute[34], the electrode mass consumed in EAF to produce 1 ton of Ni is 0.014 kg. Assuming that the electrode of a HPSR reactor degrades at the same order of magnitude than that of an electrode used in a conventional EAF [34], we can assume that all carbon from the electrode can react with oxygen from the ore, producing CO_2. Therefore, the total CO_2 emissions produced over the course of HPSR can be calculated following this chemical reaction: $C + O_2 \rightarrow CO_2$.

This means that for every mole of carbon, 1 mole of CO_2 is produced. By performing the mass balance calculations, it can be determined that 0.014 kg of C produces 1.165 moles of CO_2, a value that translates into 0.051 kg of CO_2. This accounts for only 0.11 % of the total CO_2 emissions (45 tons of CO_2/ton Ni) that RK-EF route currently emits.

Therefore, total CO_2 emissions from HPSR are estimated to be:

$$\begin{aligned}\text{Total } CO_2 \text{ emissions} \\ &= 4.44\% \text{ from mining } + 6.66\% \text{ from ore preparation} \\ &+ 0.11\% \text{ from electrode consumption } + 3.57\% \text{ from waste disposal} \\ &+ 1.11\% \text{ from transport.}\end{aligned}$$

= 15.89 % of the total CO_2 emissions emitted by RK-EF route

Therefore, hydrogen plasma processing route has a potential to cut down the CO_2 emissions by up-to 84%, assuming renewable electricity and green hydrogen are utilized during the process chain.

b) Energy Comparisons

Liu, P et. al.[35], reported that in laterite processing through RK-EF route, energy distribution is as follows: rotary dryers consume 15.2%, with both rotary kilns and Electric Arc Furnaces (EAF) consuming 42.4% each of the total energy consumption. The primary functions of rotary kilns and dryers involve moisture and chemically bound water removal, alongside partial ore reduction. Despite their significance, these pre-processing steps might exhibit inefficiencies, utilizing only 46.33% of the total energy[35] (consumed by rotary kilns and rotary dryers) for essential chemical/phase transformations and material heating—representing the minimum energy required for optimal process functionality. Energy dissipation occurs through various avenues, including losses from flue gases, dust dispersion (the dust dispersed out is at high temperature (~400-800 °C), hence taking energy with it), furnace body inefficiencies, and notably, a 15% loss during material transfer between rotary kilns and EAF, as extensively discussed in ref. [35]. The total losses occurring during the Calcination accounts for about 18.2 % of the total energy required for the entire process, as detailed in Table S3.

Table S3. Input energy distribution in rotary dryers and rotary kilns (data adapted from Ref.[35]).

Processing Step	Energy input (% of total energy consumed by whole process)	Energy utilized to run the process	Losses	Reutilized energy
**(a) Rotary Dryer**	15.2	9.96	5.320	--
**(b) Rotary Kiln**	42.42	16.83	18.2	6.68
**Total (a) + (b)**	57.62	26.79	24.08	6.68

As a solution for enhanced efficiency, direct ore (dried) processing through HPSR can theoretically avoid all previously identified losses during calcination step. This approach has the potential to render the entire process up to 18.2 % more energy-efficient, presenting a significant stride toward energy conservation and sustainable Ni extraction from laterites. Moreover, hydrogen plasma reduction exhibits faster kinetics compared to carbothermic reduction[33] and the

final product does not require refining treatments for the removal of impurities such as Si, P, C, S etc. This suggests a shorter processing time compared to the conventional rotary kiln-electric furnace (RK-EF) route, potentially leading to increased energy savings. While precise numerical projections are challenging, the proposed hydrogen plasma processing of nickel laterites emerges as a theoretically energy-efficient and environmentally friendly solution. This approach holds promise for addressing the ecological impact associated with nickel laterite production, positioning it as a sustainable alternative.

Experiments with molecular hydrogen

To evaluate the effectiveness and necessity of using hydrogen plasma species for the reduction of nickel ore, a comparative study was conducted by exposing the liquid ore to molecular hydrogen. The schematic of the reactor is shown in supplementary Fig.3 (a). A 10 g sample was melted in an induction furnace under an inert atmosphere. Once melted, a ceramic tube was inserted into the molten sample, and a continuous flow of a gas mixture containing Ar-10% H_2 was introduced for a specified duration. The melting process was observed through a glass window, revealing that gas injection into the melt caused some turbulence and stirring, although less intense than that caused by a plasma arc.

For the comparative study, the gas flow was terminated after 4 min, and the sample was allowed to solidify. The crucible was hammered to extract the solidified sample, as depicted in supplementary Fig. 3. Metallic droplets approximately 1 mm in size were observed on the surface of the sample (supplementary Fig. 3c). The sample was subsequently powdered, and the metallic droplets were recovered and weighed. These droplets were further analyzed using SEM-EDS to determine their composition. The analysis revealed that after 4 min of exposure to molecular hydrogen, only 0.3 wt.% Ni was recovered. In contrast, the same exposure time (4min) to hydrogen plasma resulted in approximately 65% nickel recovery. Extending the reduction with molecular H_2 to 10 min yielded only 4 wt.% Ni recovery.

These findings provide significant evidence that during hydrogen plasma smelting reduction (HPSR), hydrogen plasma species actively participate in the reduction process, leading to enhanced reaction kinetics.

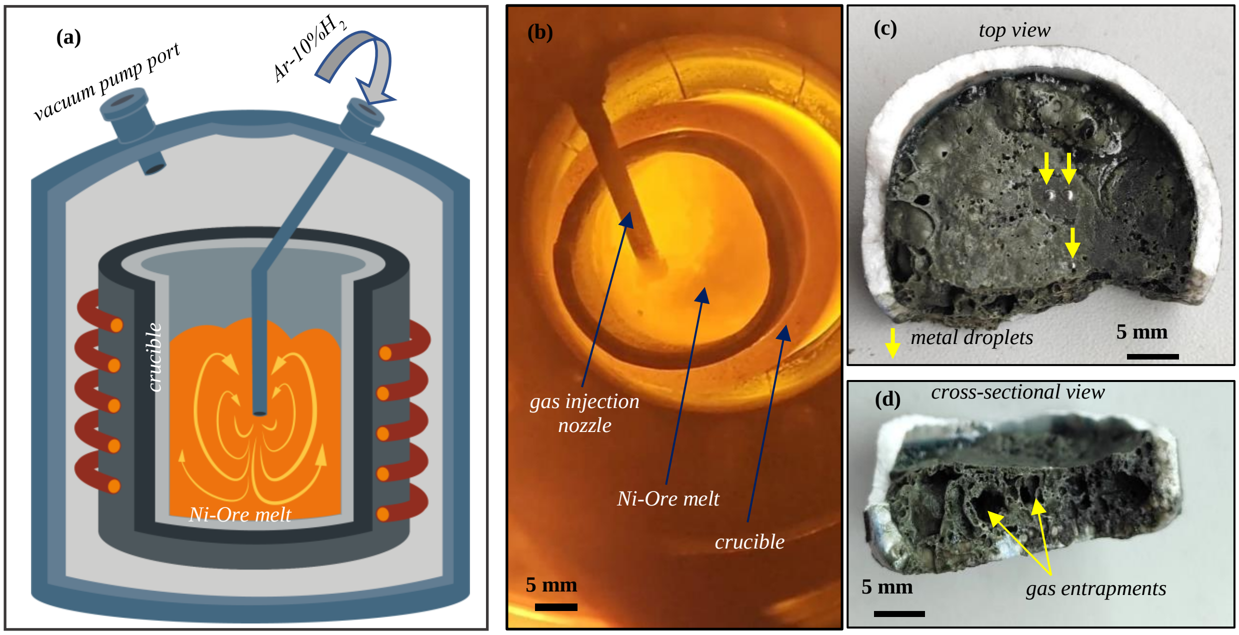

Supplementary Fig. 3: Experiments with molecular hydrogen (a) Schematic of the experimental setup for reduction with molecular hydrogen (without using any plasma source). Hydrogen gas (H_2) is introduced into the molten material through a ceramic tube, causing turbulence in the melt, as indicated by the arrows around the tube. **(b)** Image captured through a viewing port during the process, showing the molten Ni-ore and the ceramic tube inserted into the melt. **(c)** Top view of the solidified sample after 4 minutes of reduction, with metallic droplets highlighted by yellow arrows. **(d)** Cross-sectional view of the solidified sample, showing gaseous entrapments along the section, providing evidence of turbulence and gas percolation throughout the melt volume.

Cost Analysis:

Materials and alloy composition

- Ni content in alloy = 30 wt.%
- Ni recovery from the ore = 78 wt.%
- Ni content in ore = 1.56 wt.%
- Fe content in alloy = 70 wt.%
- $\text{Ore required (kg)} = \frac{\text{amount of Ni in 1 ton of FeNi (Kg)}}{\text{Ni}-\text{recovery(wt.\%)} \times \text{Ore grade(wt.\% Ni)}}$

For 1 ton of alloy

- Weight of Ni in alloy = 0.3 × 1000 = 300 kg
- Weight of Fe in alloy = 0.7 × 1000 = 700 kg

Ni ore required to produce 1 ton of alloy (300 kg Ni) = $\frac{300}{0.78 \times 0.0156}$ = 24654 kg = **24.65 ton of ore**

Oxygen removed during the process:

As explained in section "Phase transformation and reduction mechanisms"

upon melting, the olivine crystal structure breaks down in to ionic components such as:

(Fe^{2+}, Ni^{2+}, Mg^{2+}) (SiO_4^{4-}, O^{2-}) (SiO2), extended data Fig. 4:

The removal of free oxygen (O^{2-}) leads to the precipitation of metals, this means that the precipitation of Fe and Ni occurs according to the following chemical reactions:

- (Ni^{2+} + O^{2-}) + 2H = Ni + H_2O
- (Fe^{2+} + O^{2-}) + 2H = Fe + H_2O

Thus, the oxygen removed to produce 300 kg Ni is

- Moles of Ni = $\frac{300\ kg\ Fe}{58.69\ g/mol}$ = 5112.8 mol Ni
- Mass of oxygen removed = 5112.8mol O × 16g/mol = 82 kg O

The, oxygen to be removed to produce 700 Kg Fe is

- Moles of Fe = $\frac{700\ kg\ Fe}{55.85\ g/mol}$ = 12533.5 mol Fe
- Mass of oxygen removed = 12533.5 mol O × 16g/mol = 200 kg O

Total oxygen removed to make 1 ton of alloy is = 82 kg O + 200kg O = **282 kg O**

Hydrogen Requirement:

For the removal 1mol of O, 2mol of H are required. (O^{2-} + 2H + $2e^-$= H_2O)

Total moles of O removed = $\frac{282\ kg\ O}{16\ g/mol}$ = 17625 mol O

Hydrogen required = 17625 mol O × 2g/mol H_2 = 35250 g H_2 = **35 kg H_2**.

Energy requirements/ Heat balance

The energy required for the process is calculated in three steps:

- The initial step in the process involves calculating the heat and energy required to effectively treat the nickel ore. This encompasses three key phases: drying the ore, heating it to its melting point, and finally melting the ore. The specific energy requirements for

these operations were derived from differential scanning calorimetry (DSC) measurements conducted on ten different laterite ores. An average value from these measurements was utilized for the calculations.

- Second step is the energy/heat required for the reduction reactions to produce Ni-Fe alloy based on the stoichiometric calculations to produce 1 ton of alloy. The energy for individual reactions was calculated using Factsage software package using the reaction module and FactPs database.
- Third step involves the energy losses that occur in the EAF like water cooling losses, furnace body heat losses, heat lost through flue gases, and electric losses. The values are taken from Ref[35].

Energy required for drying, heating, and melting (E_m):

The specific energy required to process 1g of Ni-laterite ores, as determined from DSC measurements, is 3943 ± 276 J/g. This translates to an average value of 1095 ± 76 kWh/ton. This value aligns with the findings of O. Polyakov et al.[53], who reported that 810 kWh is required for heating and melting one ton of dry ore. Additionally, M. Jovičević-Klug et al.[31] reported that 302 kWh of energy is needed to remove 30 wt.% moisture from one ton of red mud.
Considering that Ni ores typically contain 20-30 wt.% free moisture, the combined energy requirement (810 kWh + 302 kWh = 1112 kWh) is consistent with the average value obtained from our DSC measurements (1095 kWh). This comparison suggests that the measured energy value appropriately accounts for both the energy needed for drying and for heating and melting the ore.

The energy required for drying heating and melting is calculated as follows:

$$\boldsymbol{E_m} = 1095 \times \text{total ore required per ton FeNi (kwh)}$$

Energy required for chemical reactions (E_r):

The energy consumed by chemical reactions (Er) was calculated using the thermodynamic Factsage software coupled with the reaction module and the database "FactPs". The reactions are assumed to occur in the form of two steps:

1. Conversion of H_2 to plasma species:
    - $H_2 = 2H/H^+/H^*$, (ΔH_1)
2. Reduction reaction between metal oxides and plasma species:
    - $M^{2+} + O^{2-} + 2H/H^+/H^* = M + H_2O$, ($\Delta H_2$)

The net enthalpy change of the reaction is

$$\Delta H_r = \Delta H_1 + \Delta H_2$$

The overall reaction can be written as:

$H_2 + M^{2+} + O^{2-} + 2H/H+/H^* = 2H/H+/H^* + M + H_2O$

It can be re-arranged as:

$(M^{2+} + O^{2-}) + H_2 = M + H_2O$

For $M^{2+} = Ni^{2+}$ and Fe^{2+}, at 1500°C

- $(Ni^{2+} + O^{2-}) + H_2 = Ni + H_2O$ (*$\Delta H_{r,Ni}$ = -3.030 kj/mol Ni)*
- $(Fe^{2+} + O^{2-}) + H_2 = Ni + H_2O$ (*$\Delta H_{r,Fe}$ = -21.475 kj/mol Fe)*

Therefore, the energy required to produce 300 kg (5112.8 mol) of Ni:

5112.8 mol Ni × (-3.03 kJ/mol Ni) = -15.9 MJ

Energy required to produce 700 kg (12533.5) of Fe:

12533.5 mol Fe × (-21.47 kJ/mol Ni) = -269.1 MJ

The means that the total energy consumed for chemical reactions (***E_r***):

= (-15.9 MJ) + (-269.1 MJ) = **-285 MJ,**

which can be translated into **-79.13 kWh.**

Energy losses in EAF (E_l):

During Electric Arc Furnace (EAF) operation, energy losses are inevitable. They include furnace heat loss, heat loss to off-gases, cooling water losses, and electrical losses. These losses are accounted for in the calculations, with typical values sourced from ref[35].

Therefore, the total energy (***E_t)*** consumed during the process is calculated as follows:

$$\boldsymbol{E_t = E_m + E_r + E_l}$$

Where, $\boldsymbol{E_m}$ stands for the energy utilized to dry, heat and melt of the ore, $\boldsymbol{E_r}$ stands for the energy utilized by chemical reactions and $\boldsymbol{E_l}$ stands for the energy losses occurring during EAF operation.

Argon (Ar) gas required:

During the process the Ar gas requirement was calculated by assuming a 100 ton standard furnace with dimension of 16 m in diameter and 1.5 m height of gas compartment[31,59]. The gas mixture employed contains 90% Ar, 10% H_2. This means that the volume of Ar gas in the furnace will be as follows:

$$\text{Volume of Ar (V}Ar) = 0.9 \times \text{volume of the gas compartment (V}g)$$

Assuming the gas density of Ar at ambient conditions is 1.69 kg/m^3, the total mass of Ar required is as follows:

$$Mass\ of\ Ar\ (kg) = V_{Ar}\ (m^3) \times\ 1.69\ (Kg/m^3).$$

Cost Calculations:

The costs of these different parameters used in the current study were sourced from the references provided. Table. S4 presents the values as given directly by the references. To ensure consistency in comparison, all cost figures were normalized to a per-ton ore basis. This adjustment accounts for differences in recovery rates, alloy grade, and other process parameters between the reference data and the current study. The normalization allows the integration of the reference values into the calculations based on the specific recovery rates, feed processed, and alloy grades used in this study.

Table. S4: Cost and the variables used in this study: values adapted from *Ref.*[31,54,55,56,57,58.]

Description	Value [units]
Price of argon gas[31,59]	0.931 [EUR/kg]
Price of hydrogen gas[31,59]	3.30 & 5 [EUR/kg]
Price of electricity[31,59]	50 & 100 [EUR/MWh]
Price of labour[31,59]	40.23 [EUR/ton ore]
The depreciation fee (assuming a 15-year lifespan for the equipment) [54]	7 EUR/ton alloy*
Equipment costs[54]	26 EUR/ton alloy*

Infrastructure costs (assuming a 40-year lifespan for the infrastructure)[54]	10 EUR/ton alloy*
Price of alloys for EAF[31,59]	1777 [EUR/t]
Consumption of alloys for EAF[31,59]	7.77 [kg/ton ore]
Price of electrodes[31,59]	4000 [EUR/t]
Consumption of electrodes[31,59]	1.415 [kg/ton ore]
Transportation costs[54]	116 EUR/ton alloy*
Waste water treatment costs[54]	0.2 EUR/ton alloy*
Solid waste to landfill costs[54]	13.5 EUR/ton alloy*
Costs of maintenance[54]	16 EUR/ton alloy*
Energy required for drying, heating and melting the ore (E_m)	3943+/-276 J/g
Ni ore cost (1.5wt.% Ni)[56]	40 EUR/ton
Selling price Ni (averaged over last three years) [58]	20752 [EUR/t]
Selling price of residual slags (SP_{slag}) [55]	23 [EUR/t]

***** *values adapted from Ref. [54], the study selected one ton of ferronickel as the functional unit. The ore burden used in the life cycle inventory to produce one ton of alloy is 12.67 tons. The values in the cost analysis of this study are normalized to per-ton ore basis.*

The capital expenditures ***CAPEX*** of the Electric Arc Furnace (EAF) used to perform the hydrogen plasma smelting reduction are calculated on a per-ton basis of ferronickel, taken from Ref[54]. This includes

- The depreciation cost (assuming a 15-year lifespan for the equipment)
- Equipment costs
- Infrastructure costs (assuming a 40-year lifespan for the infrastructure).

The total operational expenses ***OPEX*** include:

- Ore costs
- Costs of gases used (Ar and H_2)
- Costs of total energy used (E_t)
- Maintenance costs
- Labor costs
- Costs of alloys used in EAF
- Transportation costs
- Waste water treatment costs
- Solid waste to landfill costs
- Costs of electrodes consumed during furnace operation.

Selling value of residual slags (V_{slag}):

The slags produced during the Hydrogen Plasma Smelting Reduction (HPSR) process are primarily composed of magnesium silicates (see Fig. 2a of the manuscript), which exhibit potential for use in road construction materials. Given their composition, these slags were included in the economic evaluation. Based on the mass balance from our lab-scale setup, approximately 40 wt.% of the ore feed was recovered as slag. The potential capital profit from selling the slag was calculated using the following equation:

$$V_{slag} = \ Amount\ of\ ore\ (ton) \times 0.4\ \times\ SP_{slag}$$

Market price when selling FeNi:

The industry projections suggest that nickel prices are expected to experience a significant increase by 2030, driven by the rising demand of the electric vehicle (EV) sector, the energy storage industry, and expanding green technologies. According to reports by authoritative market analysts and agencies, such as the International Nickel Study Group (INSG) and various market research firms [57] this upward trend is anticipated due to both supply constraints and the accelerated transition to renewable energy sources.

The market fluctuations over past three years are high because Ni is subject to massive global market speculations, as shown in the supplementary Fig 4: (whose data was adapted from London metal exchange)[58]. To account for these fluctuations, the selling price of ferronickel is here determined based on the average price observed over the past three years. This approach helps mitigate the impact of short-term volatility and provides a more stable pricing strategy. The price of ferronickel is calculated by multiplying the average price (20752 EUR/ton) with the Ni content (30 wt.%) per ton alloy.

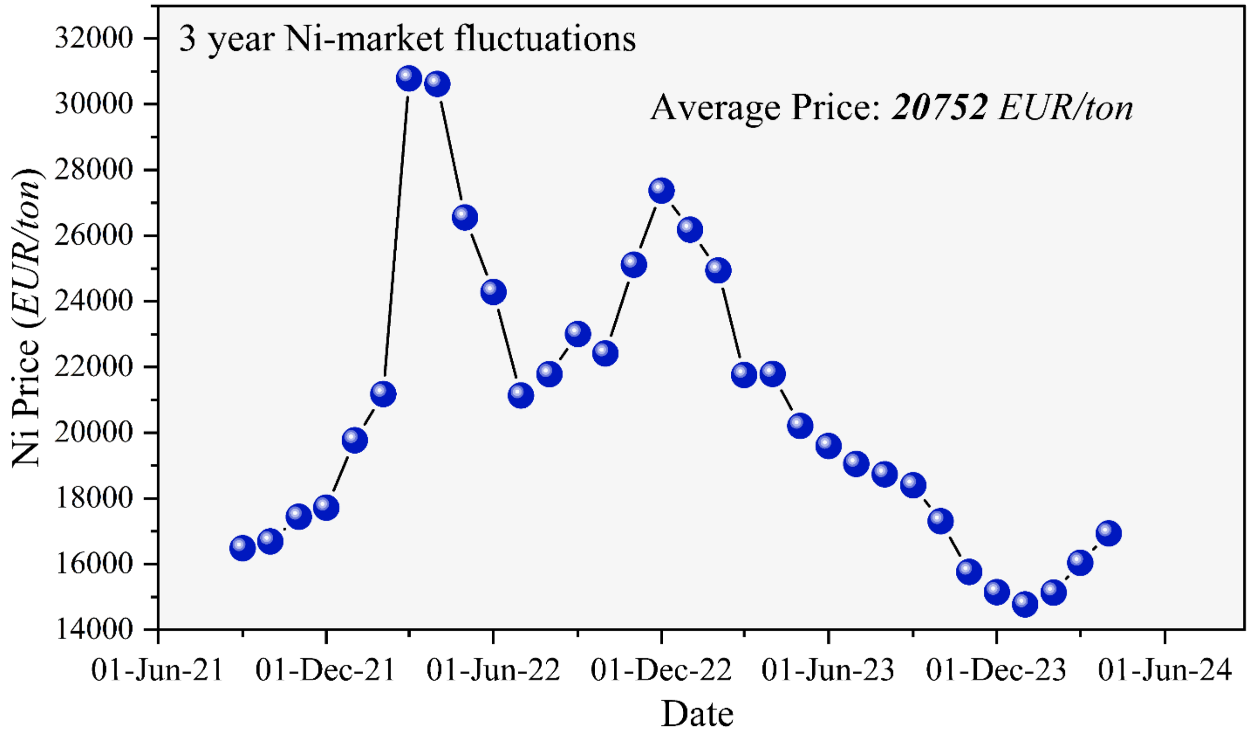

Supplementary Fig. 4: Ni-market variation over last three years (data taken from London metal exchange)[58].

Final capital outcome *(FO):*

The Final capital outcome of the process was calculated by using the formula

$$FO = selling\ price\ of\ FeNi\ -\ OPEX - CAPEX\ +\ V_{slag}$$

The final capital outcome of the process is evaluated as a function of ore grade variability, as illustrated in Supplementary Fig. 5. While the respective ore grade tends to have a slight positive correlation with the economic viability of the process, the financial outcomes do not fluctuate as significantly as one might expect. This can be attributed to the interplay between ore costs and processing efficiency.

As ore grades increase, the mass of material required to produce one ton of alloy decreases, leading to reductions in key cost components such as labor, raw material input, energy consumption, and electrode wear. However, the cost of Ni-ore also increases with higher grades. Data obtained from Ref.[56] reveals that the cost of nickel ore varies with grade, with known values of 24 EUR/t, 26 EUR/t, 33 EUR/t, 39 EUR/t, and 62 EUR/t at 1.2 wt.% Ni, 1.3 wt.% Ni, 1.4 wt.% Ni, 1.5 wt.% Ni, and 1.8 wt.% Ni content, respectively. For Ni grades where explicit data were unavailable, a linear

interpolation method was employed to estimate the prices, and these interpolated values were utilized for subsequent cost analysis calculations.

Consequently, while increasing the ore grades lead to a reduced material burden and lower energy consumption, the corresponding rise in ore costs at higher grades mitigates some of the capital gains achieved through energy savings. This intricate balance helps maintain the capital outcome at approximately 2500 EUR/ton FeNi, reflecting a steady financial performance despite the variations in ore grade. Therefore, the economic impact of ore grade is nuanced; although higher grades improve processing efficiency, the increased costs associated with high-grade ores play a critical role in shaping the overall capital outcome of the process.

To further quantify the cost dynamics, we analyzed the process under two energy sourcing scenarios as upper and, respectively, lower bound cases: grey hydrogen (3.30 EUR/kg)[31,59] paired with low-cost electricity (50 EUR/MWh) [31,59] and green hydrogen (5 EUR/kg)59 combined with more expensive renewable electricity (100 EUR/MWh)[31,59]. The corresponding results are illustrated in Supplementary Fig. 5, with grey bars representing the former scenario and green bars the latter. In both cases, the financial outcomes remain favorable, demonstrating the robustness of the process under varying energy inputs. These findings indicate not only the economic viability of hydrogen plasma smelting, but also its potential for significant environmental advantages, particularly in decarbonizing metallurgical processes through the integration of green hydrogen and renewable energy.

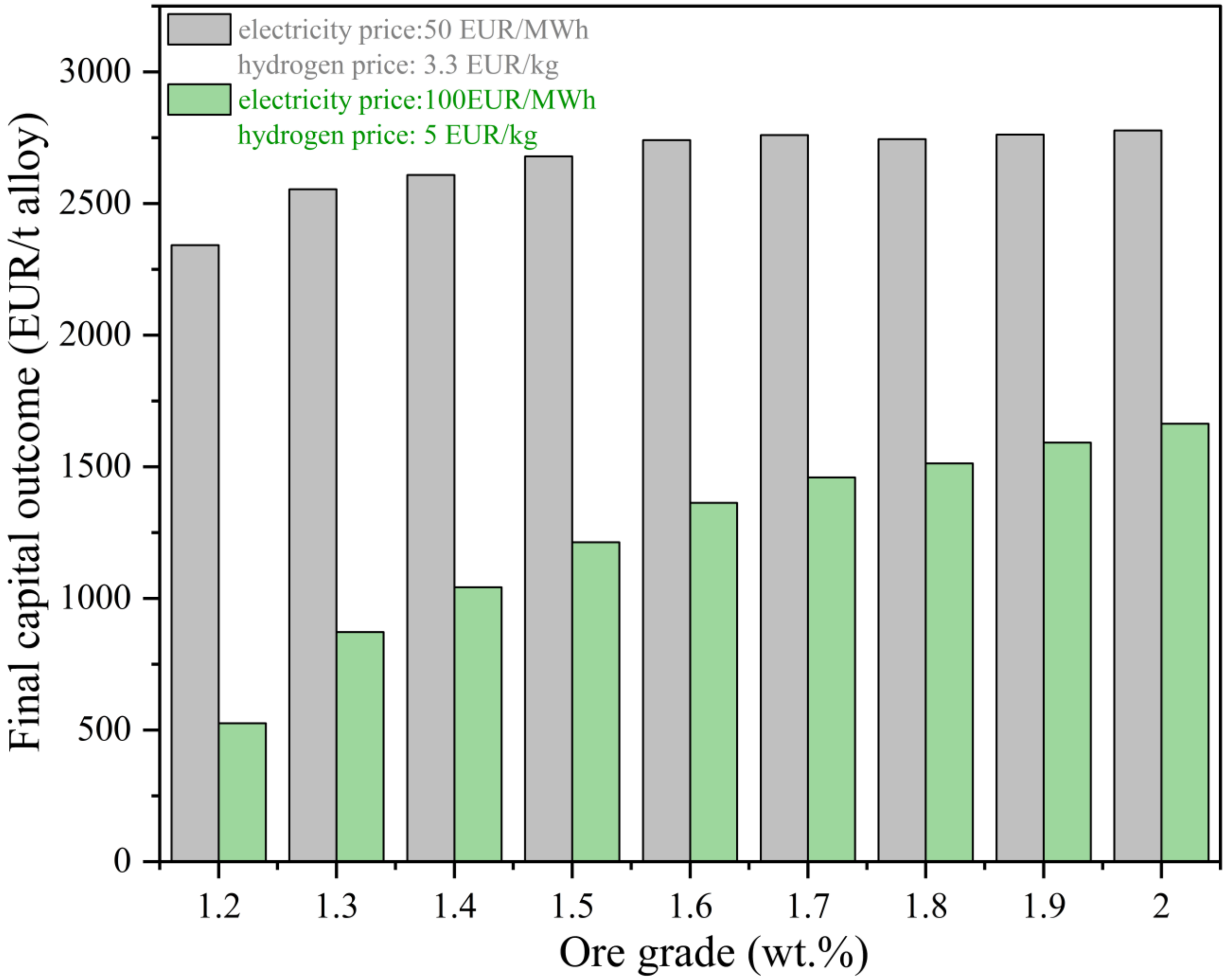

Supplementary Fig. 5: Financial capital outcome of the process as a function of ore grade. The grey bars represent the financial capital outcome when utilizing low-cost electricity (50 EUR/MWh) and grey hydrogen (3.3 EUR/kg). The green bars depict the financial capital outcome when using more expensive renewable electricity (100 EUR/MWh) and green hydrogen (5 EUR/kg).

References:

43. Whittington, B. I. & Muir, D. Pressure Acid Leaching of Nickel Laterites: A Review. *Miner. Process. Extr. Metall. Rev.* **21**, 527–599 (2000).
44. Agatzini-Leonardou, S. *et al.* Heap Leaching of Greek Low-Grade Nickel Oxide Ores by Dilute Sulphuric Acid at a Pilot-Plant Scale. in *International Conference on Raw Materials and Circular Economy* 65 (MDPI, 2021). doi:10.3390/materproc2021005065.
45. Luo, J. *et al.* Control of slag formation in the electric furnace smelting of ferronickel for an energy-saving production. *J. Clean. Prod.* **287**, 125082 (2021).
46. Gu, F. *et al.* Facile Route for Preparing Refractory Materials from Ferronickel Slag with Addition of Magnesia. *ACS Sustain. Chem. Eng.* **6**, 4880–4889 (2018).
47. Wei, W., Samuelsson, P. B., Tilliander, A., Gyllenram, R. & Jönsson, P. G. Energy consumption and greenhouse gas emissions of nickel products. Energies (Basel) **13**, (2020).
48. Eliott, R. S. B. A Study on the Role of Sulfur in Thermal Upgrading of Nickeliferous Laterite Ores. (Queen's University, 2015).
49. Sherritt. Does matte matter? (2021).
50. Sun, W., Li, X., Liu, R., Zhai, Q. & Li, J. Recovery of Valuable Metals from Nickel Smelting Slag Based on Reduction and Sulfurization Modification. *Minerals* **11**, 1022 (2021).
51. Dhawan, N., Manzoor, U. & Agrawal, S. Hydrogen reduction of low-grade banded iron ore. Miner Eng **187**, 107794 (2022).
52. Kim, S. H. et al. Influence of microstructure and atomic-scale chemistry on the direct reduction of iron ore with hydrogen at 700°C. Acta Mater 212, 116933 (2021).
53. Polyakov, O. Technology of Ferronickel. in Handbook of Ferroalloys: Theory and Technology 367–375 (Elsevier, 2013). doi:10.1016/B978-0-08-097753-9.00010-1.
54. Ma, X. et al. International Journal of Life Cycle Assessment **24**, 1840–1850 (2019)
55. Chowdhury, S. R. Recycled Smelter Slags for In Situ and Ex Situ Water and Wastewater Treatment—Current Knowledge and Opportunities. *Processes* vol. 11 Preprint at https://doi.org/10.3390/pr11030783 (2023).
56. Metal.com. Nickel price. *Metal.com*. https://www.metal.com/Nickel (accessed October 10, 2024).
57. Fitch Solutions. Beyond 2024: Nickel prices to increase steadily to 2028. *MINING.COM*. https://www.mining.com/beyond-2024-nickel-prices-to-increase-steadily-to-2028-fitch/ (accessed October 10, 2024).
58. London Metal Exchange. LME Nickel. *https://www.lme.com/Metals/Non-ferrous/LME-Nickel*
59. Vogl, V., Åhman, M. & Nilsson, L. J. Assessment of hydrogen direct reduction for fossil-free steelmaking. *J Clean Prod* **203**, 736–745 (2018)
